# The psychology of criminal authority: Introducing the Legitimacy of Secret Power Scale

**DOI:** 10.1177/13684302241290935

**Published:** 2024-12-08

**Authors:** Giovanni A. Travaglino, Alberto Mirisola, Chanki Moon, Pascal Burgmer, Hirotaka Imada, Isabella Giammusso, Silvana D’Ottone, Kengo Nawata, Miki Ozeki, Dominic Abrams

**Affiliations:** 1 Royal Holloway University of London, UK; 2 University of Palermo, Italy; 3 University of Southampton, UK; 4 Pontificia Universidad Católica de Chile, Chile; 5 Fukuoka University, Japan; 6 Okayama University, Japan; 7 University of Kent, UK

**Keywords:** authority, governance, legitimacy, organized crime, secret power

## Abstract

The state’s monopoly on sovereignty can be challenged by criminal systems capable of gaining legitimacy within communities. Understanding the psychological basis of such legitimacy requires broadening traditional conceptualizations of authority to consider how it operates without legal backing and outside formal channels. This research introduces the Legitimacy of Secret Power (L-SP) Scale, a tool measuring individuals’ appraisal of illegal groups’ power. We validated L-SP through three studies (*N*_total_ = 3,173). Findings demonstrate a reliable, 20-item mono-factorial structure. Study 3 tested L-SP’s measurement invariance in the UK, Italy, Japan, and the US. Across studies, L-SP correlated with support for illegality, ideologies of masculine honor, and social dominance. It was inversely related to the perceived national threat of criminal groups, democratic attitudes, and police legitimacy. Notably, L-SP predicted individuals’ willingness to report criminal groups independently of their fear of these groups or perceptions of police legitimacy. Theoretical implications and future directions are discussed.

In modern societies, the state formally has a monopoly on governance functions, such as using force to maintain social order and allocating resources. However, state sovereignty may be challenged by the presence of criminal systems of power (N. Barnes, 2017; Travaglino & Abrams, 2019). The existence of such systems carries significant implications for security, democracy, and justice (Travaglino et al., 2023). A crucial scientific challenge is to investigate the psychological factors contributing to their persistence.

Although secretive and violent (N. Barnes, 2017), the ability of criminal systems to control territories and inhibit opposition from communities cannot be sustained solely by coercion. It is our contention that, like other forms of authority, criminal systems need to establish a degree of legitimacy among the individuals they intend to influence. Intracultural appropriation theory (ICAT) proposes key psychological dynamics that underpin positive attitudes toward criminal organizations in Southern Italy (Travaglino & Abrams, 2019). Italian criminal organizations are impactful groups establishing systems of parallel governance and acting as a “state within the state.” Their ability to cultivate legitimacy facilitates their illegal activities within communities, making it more challenging for legal authorities to combat them.

However, criminal systems of various dimensions and degrees of impact have emerged in many communities worldwide (Lessing, 2020), and the question remains as to whether the psychological dynamics proposed by ICAT are generalizable across contexts and types of groups. To address this question, a necessary first step is to develop a reliable measure for tapping into individuals’ appraisals of criminal systems of power across settings. Gaining a better understanding of such dynamics might contribute to generating more comprehensive models of how authority operates when wielded outside formal institutions and without legal sanctioning. This article reports on three studies developing and validating the Legitimacy of Secret Power (L-SP) Scale.

## Authority, Legitimacy, and Secret Power

The philosopher Thomas Hobbes (1651/2017) and the social theorist Max Weber (1922/2019) were among the thinkers arguing that establishing and maintaining a system of social order—the set of norms and laws that make social life possible—requires the presence of a hierarchy characterized by a smaller social entity exerting power over a larger one (see Hechter & Horne, 2009). This arrangement may be advantageous to the collective because, in the long run, it reduces interpersonal violence and enables individuals to cooperate and achieve essential goals (Lautenbacher & Fritsche, 2023). However, it is also susceptible to tension and conflict, primarily because of the inequity inherent to the relationship between power holders and the powerless.

This tension can be resolved by means of coercion, where power is harnessed (often violently) to compel obedience. However, the sole reliance on coercive means is highly unstable, unproductive, and likely to provoke resistance (Turner, 2005). From the power holders’ perspective, a more effective strategy is to cultivate a belief in the legitimacy of their power (Beetham, 2013; Zelditch, 2006). Legitimacy is a complex and multifaceted concept (Fukuyama, 2012; Hechter, 2009; C. Johnson et al., 2006; Tyler, 2006; Zelditch, 2001). It encompasses the appraisal of various forms of social organization, including actions, persons, groups, regimes, and systems. Generally, something is legitimate if “it is in accord with the norms, values, beliefs, practices, and procedures accepted by a group” (Zelditch, 2001, p. 33). Legitimate power (or authority) is acknowledged as rightfully held, even by those who would benefit from alternative forms of social organization (Zelditch, 2006). Thus, legitimacy contributes to the stability of power structures and reduces the likelihood of opposition by grounding obedience in people’s sense of duty and obligation.

In the context of governance, social psychology offers various models of how legitimacy operates (Jost & Major, 2001). Theories such as system justification (Jost, 2019) and social dominance (Sidanius & Pratto, 2001; Sidanius et al., 2004) suggest the existence of the tendency among people to justify the status quo and accept social hierarchies. System justification theory asserts that fundamental psychological needs—reducing uncertainty, alleviating insecurity and threat, and facilitating social coordination—drive this acceptance, potentially to individuals’ own detriment. Social dominance theory focuses on individual variations in preferences for hierarchical social structures. This theory posits that people have differing orientations towards inequality, with some having a stronger preference for hierarchical relationships where certain groups, typically their own, dominate others.

Additionally, procedural justice theory anchors perceptions of legitimacy in authorities’ adherence to principles of fair treatment and just procedures (Jackson et al., 2013; Tyler, 2006). Institutions and legal bodies are more likely to be perceived as legitimate when they demonstrate fairness in their decision-making processes and their interactions with the public. Authorities’ fairness signals respect and a sense of inclusion to individuals within the group, enhancing people’s voluntary compliance (Tyler & Blader, 2003). The focus on the fairness of procedures does not imply that individuals overlook other aspects of authorities’ behavior—for instance, how they allocate resources. Nevertheless, research indicates that judgments about resource allocations are shaped by the perceived fairness of procedures (Tyler & Blader, 2000). Moreover, the perceived legitimacy of the authority (Tyler, 2006) or factors that contribute to the legitimacy of the allocation decision (e.g., decisions favoring the ingroup; Biella & Sacchi, 2018; Civai et al., 2013) can mitigate individuals’ tendencies to reject unfair decisions.

However, the question remains of how governance operates outside institutional, legal, and formal channels. Globally, parallel systems can emerge, coexisting with the state in various ways. Such systems challenge the state’s exclusive sovereignty over a territory, exerting authority across multiple key domains of social life (e.g., Strange, 1996). In other words, it is important to acknowledge that individuals live in a complex web of interconnected power relations characterized by distinct rule-makers, formal and informal institutions, and overlapping frameworks of social order.

Challenges to state sovereignty can arise from “above” in the form of supernational and global forms of governance (e.g., large organized religions, global corporations, etc.; see Strange, 1996). They can also emerge from “below,” from the criminal underworld of criminal organizations, gangs, and other illegal groups. Notably, criminal systems of power are not confined to fragile or war-torn states, where violent groups may become the primary entities capable of maintaining order (Sánchez de la Sierra, 2020). They also emerge in wealthier, more democratic, and stable nations—for instance, the UK or Italy (Lessing, 2020).

A distinctive characteristic of such systems is that they wield power while being actively criminalized, and hence invalidated, by state institutions. Moreover, because they constitute “informal” power structures, their appraisal by individuals and communities often transcends the traditional political spectrum, reflecting a complex interplay of cultural beliefs and values rather than conforming to conventional left/right orientations (Travaglino & Abrams, 2019). From a sociopsychological perspective, the emergence of criminal systems capable of establishing social order requires broadening conceptualizations of power, legitimacy, and ideology.

Travaglino and Abrams (2019) referred to the power of criminal systems as “secret power,” defined as,

[N]ot formally recognized or delimited by statutory authorities, yet it is constituted and regulated through clearly structured social properties such as group memberships, social or geographical reach, systems of exchange and responsibility, forms of constraint, and culturally rooted rules, often backed by tradition and precedent. (Travaglino & Abrams, 2019, p. 75)

This power is secret, or “hidden,” because criminal systems seek “to maximize governmental power without taking on the formal responsibilities of political rule” (Cockayne, 2016, p. 10).

Like other forms of power, secret power can last and be effective only if it can cultivate a belief in its legitimacy (Travaglino & Abrams, 2019). Research on ICAT addresses these dynamics of legitimization in the context of criminal organizations in Southern Italy (Travaglino et al., 2022). For instance, this research has demonstrated a link between individuals’ endorsement of masculine honor values and their positive appraisals of criminal organizations. Masculine honor refers to a set of values emphasizing the importance of male self-reliance and violence in response to offenses against one’s honor (C. D. Barnes et al., 2012; Rodriguez Mosquera, 2016). Criminal groups appropriate and strategically emphasize the importance of such values in their dealings with the community. This research suggests that as long as criminal groups’ use of violence adheres to specific standards, such as those embodied in masculine honor values, it can actually serve as a tool to reinforce their legitimacy (cf. Fiske & Rai, 2015). Moreover, this perspective is complemented by findings indicating that between-person differences in social dominance orientation (SDO) are also associated with the legitimization of criminal organizations (Travaglino et al., 2022).

## Overview of the Studies

A crucial question is whether the dynamics of legitimization of criminal authority proposed by ICAT are generalizable across contexts. To investigate the psychological correlates of individuals’ appraisal of secret power, a necessary first step is to build a reliable measure tapping into the perception of practices of criminal governance. To ensure scores are comparable across settings, the measure should focus on individuals’ assessments of the provision of social order and governance by criminal groups rather than evaluating specific organizations. The purpose of the present research is to establish a reliable and externally valid measure of the perceived legitimacy of secret power and to test its associations and predictive potential for theoretically specified outcomes as well as practical application. We conducted three studies. For all the studies, we report all data, measures, and participant exclusions. Analyses were conducted using R (Version 4.2.1; R Core Team, 2022). Datasets and code (including a full list of the packages used) are available on the Open Science Framework database (OSF; https://osf.io/v895f/).

## Study 1

The primary objective of Study 1 was to explore the structure of a novel L-SP scale. Starting from the notion of secret power, we specified this construct as individuals’ appraisal of the enactment of governance functions by criminal groups (Lazarsfeld, 1958). We focused on functions in domains key to governance, including the management of social relations, the regulation of justice and social order, and the administration of the economy (cf. Fukuyama, 2012). Next, we generated a set of items and employed factor analysis techniques to select the final list of indicators.

To generate our initial pool of items, we reviewed the interdisciplinary literature on various forms of criminal governance enacted by criminal organizations and other types of criminal groups across geographical areas and wrote statements for each of them (e.g., N. Barnes, 2017; Densley, 2013; Lessing, 2020; Travaglino & Abrams, 2019). We selected “the community” as the main frame because, although criminal groups affect entire nations, their ability to exert governance is typically rooted within specific territories. Criminal organizations often establish strongholds in particular neighborhoods or regions, embedding themselves into the social and cultural fabric of these areas.

Colleagues with expertise in political sciences, sociology, and psychology reviewed the items (DeVellis, 2017). After initial feedback, some items were dropped due to lack of clarity. Items that had initially contrasted the power of the government with the secret power of criminal groups (e.g., “Criminal groups are better than the police at resolving disputes among people”) were rewritten (“Criminal groups are good at resolving disputes among people”), so that the association between L-SP and individuals’ attitudes towards legal forms of power could be tested rather than implied by the form of the items. A final list of 40 items was administered to our sample.

In this study, we also measured participants’ endorsement of masculine honor (C. D. Barnes et al., 2012) and their willingness to cooperate with legal authorities to oppose criminal groups (Jackson, 2013). To provide initial evidence for the validity of the new scale, we tested L-SP’s associations with such constructs. ICAT would predict a positive association between L-SP and endorsement of masculine honor, owing to criminal groups’ appropriation and display of such values.

Compliance with legal authorities is of central importance for maintaining social order and a key demonstration of state sovereignty (Jackson, 2013). Research has so far primarily focused on the perceived legitimacy of legal authority as a predictor of compliance. Here, we complement this perspective by testing the role of people’s legitimization of secret power. We expected that stronger L-SP would be linked to a lower willingness to cooperate with legal authorities.

### Participants and Procedure

Four hundred and one British individuals participated in a study about the perception of “illegal social practices” (200 male, 199 female, one nonbinary, one did not report their gender; *M*_age_ = 40.78, *SD*_age_ = 13.83). Participants were recruited via the online platform Prolific using the survey software Qualtrics. Sample size requirements for exploratory factor analysis vary depending on a range of elements, including the communality of the items and the degree of overdetermination of the factor (Goretzko et al., 2019). Because such elements were unknown, we planned for a sample size of *N* = 400, as recommended by MacCallum et al. (1999). The study included one attention check, “This is an attention check please select agree on the scale below.” After completing the items (randomized) and providing demographic information (gender and age), participants were debriefed and compensated for their time.

### Measures

A complete list of items is available at the OSF (https://osf.io/v895f/).

#### Legitimacy of Secret Power (L-SP) Scale

The initial item pool of the L-SP scale consisted of 40 items (see Table 1). Participants indicated their level of agreement on a 7-point scale (1 = *strongly disagree*, 7 = *strongly agree*). The following instructions preceded the measure:

In many countries, there exist organized groups that operate outside the bounds of the law. These groups may include but are not limited to mafias, cartels, gangs, vigilantes, and other similar organizations that are not recognized by the government and do not comply with established legal frameworks. In the following statements, we refer to these groups as “criminal groups.” We would like your views on such groups. Please rate your level of agreement with each of the following statements.

**Table 1.** Initial pool of items for the Legitimacy of Secret Power (L-SP) Scale with standardized factor loadings.

	Items	λ
*L-SP3	Criminal groups are sometimes necessary to maintain order in the community	.78
*L-SP19	Criminal groups play a positive role in promoting economic development in disadvantaged communities	.77
*L-SP17	Criminal groups improve the local economy	.75
*L-SP27	Criminal groups are necessary to get things done in the community	.73
*L-SP2	Criminal groups provide protection in an efficient way in the community	.72
*L-SP28	Criminal groups can sometimes be trusted to act in the community’s best interests	.72
*L-SP32	Criminal groups are effective at maintaining social stability	.72
*L-SP1	Criminal groups provide justice effectively in the community	.71
*L-SP5	Criminal groups protect individuals’ rights	.71
*L-SP30	Criminal groups have knowledge and understanding of local customs and traditions, which allows them to serve the community well	.70
*L-SP4	Criminal groups are good at resolving disputes among people	.69
*L-SP7	In some cases, it is necessary to rely on criminal groups for security	.68
*L-SP38	The use of force by criminal groups is sometimes justified	.67
*L-SP40	Criminal groups should serve as the community’s representatives	.67
*L-SP24	Criminal groups invest in development projects for vulnerable communities	.67
L-SP16	Criminal groups are responsive to the economic needs of the community	.65
L-SP9	Criminal groups provide a sense of protection for vulnerable individuals who might otherwise be victimized by the more powerful	.64
L-SP6	The use of violence by criminal groups to punish wrongdoings is sometimes justified if it is in the community’s best interests	.63
L-SP29	The existence of criminal groups is a necessary evil in societies	.63
*L-SP22_R	Criminal groups harm the economy of the community	.61
L-SP20	Criminal groups are a legitimate response to an immoral economy	.59
L-SP18	Criminal groups often provide economic opportunities for disadvantaged communities	.58
*L-SP10_R	Criminal groups’ activities can only have negative consequences for people	.57
L-SP31	Criminal groups are responsive to the community because they are intimately connected with it	.57
L-SP23	Criminal groups provide employment opportunities that might not be otherwise available	.55
L-SP39	Criminal groups serve as a form of resistance against oppressive systems of power	.53
*L-SP21_R	Criminal groups exploit the community for their own financial gain	.52
L-SP33	For individuals, it is better to negotiate with criminal groups than to combatting them	.46
L-SP15	Criminal groups provide opportunities for economic advancement that are not available through legal means	.45
*L-SP13_R	People in areas with criminal groups live in fear due to criminal groups’ arbitrary justice	.44
*L-SP34_R	The presence of criminal groups creates conflicts within communities	.44
L-SP8	Criminal groups are effective at enforcing rules	.42
L-SP12_R	The rules imposed by criminal groups lack accountability	.39
L-SP26_R	Criminal groups deter legitimate businesses from operating in the community	.38
L-SP14_R	Criminal groups neglect individual rights when enforcing the rules	.37
L-SP25_R	The economic activities of criminal groups lead to economic instability for the community	.37
L-SP35_R	Criminal groups uphold traditions that benefit only their interests rather than the broader community	.19
L-SP11_R	The absence of formal procedures leads to unfair outcomes when criminal groups resolve disputes among people	.17
L-SP36_R	Community involvement with criminal groups leads to social stigmatization	.17
L-SP37_R	Criminal groups use local culture as a tool to justify their harmful activities	.09

*Note.* *Indicate the items retained in the final version of the L-SP scale. Scale instructions are described in the Measures section.

#### Masculine honor ideology

Endorsement of masculine honor values was measured employing four items drawn from C. D. Barnes et al.’s (2012) Honor Ideology for Manhood Scale (e.g., “A man has the right to act with physical aggression toward another man who slanders his family”; 1 = *strongly disagree*, 7 = *strongly agree*; α = .80).

#### Willingness to cooperate with legal authorities

Cooperation intentions were measured using the item (Jackson, 2013), “How likely are you to inform the police if you become aware of a crime committed by a criminal group?” (1 = *not at all likely*, 7 = *extremely likely*).

### Results and Discussion

Four participants were excluded from the analyses because they failed the attention check. We determined the number of factors to retain using a parallel analysis (Horn, 1965; Lim & Jahng, 2019). Parallel analysis tends to produce a more accurate estimation than other dimensionality assessment methods (Crawford et al., 2010). This technique compares the eigenvalues from randomly generated data to those based on sample data. Factors whose eigenvalues are larger than the eigenvalues from the randomly generated data are retained. Results suggested the presence of two factors.

We cross-checked this result using additional factor retention techniques. Specifically, we employed a sequential approach using the lower bound of the 90% confidence interval of the root mean square error of approximation (RMSEA; Preacher et al., 2013), the Hull method based on the comparative fit index (CFI; Lorenzo-Seva et al., 2011), and the comparison data approach (Ruscio & Roche, 2012). While none of these techniques consistently outperform the others across different data characteristics (for additional details, see Auerswald & Moshagen, 2019; Preacher et al., 2013), convergence in the results would strengthen confidence in the model structure. All techniques suggested the presence of two factors, consistent with the results of the parallel analysis.

We conducted an exploratory factor analysis with promax rotation and principal axis factoring. Upon inspecting the loadings, it was observed that the second factor predominantly captured the reversed items, suggesting that it may be a methodological artifact rather than a reflection of an underlying construct. Therefore, we modeled a single-factor solution using maximum likelihood estimation with standard errors robust to nonnormality (Huber, 1967). We evaluated model fit using three indices (Kline, 2016) and the following cut-off values (Schermelleh-Engel et al., 2003): CFI > .95, standardized root mean square residual (SRMR) < .10, and RMSEA < .08. Residuals of the reversed items were allowed to covary. This approach produces better and more stable estimates compared to alternative modeling techniques employing a distinct (method) factor (Marsh, 1989; Marsh & Bailey, 1991). The model fit was acceptable, CFI = .90, RMSEA = .05, 90% CI [0.05, 0.06], SRMR = .06, although the CFI indicated some degree of misspecification. Standardized factor loadings are summarized in Table 1.

This model was employed to reduce the number of items. We aimed to maintain a robust measurement model of the construct while ensuring a more manageable length for both researchers and respondents. We selected the 15 items with the highest factor loadings. Moreover, we retained the five highest-loading reverse items to mitigate the potential for acquiescence bias in cross-national studies. The final set of 20 items covered a range of topics, including the management of order (L-SP3), justice (L-SP1), and economy (L-SP17). Only five reversed items were selected, consistent with our aim to focus on the legitimization of secret power rather than its rejection. The 20-item scale had excellent fit, CFI = .96, RMSEA = .05, 90% CI [0.04, 0.06], SRMR = .04, and internal reliability, α = .94 (factor loadings are summarized in Tables A and B, Supplemental Material).

Table 2 summarizes the correlations, means, and standard deviations for the final version of the scale (exact *p* values are presented in Table C, Supplemental Material). The values of skewness (0.31) and excess kurtosis (−0.61) of the L-SP scale indicated a departure from normality (reference values 0 and 3, respectively), with a positively skewed and leptokurtic distribution.

**Table 2.** Means, standard deviations, and correlations: Study 1.

	1	2	3	4	5	*M*	*SD*
(1) L-SP						2.51	0.93
(2) Masculine honor	.26*** [0.16, 0.35]					2.94	1.28
(3) Willingness to report criminal groups activities	−.35*** [−0.44, −0.26]	−.16** [−0.25, −0.06]				4.27	1.79
(4) Age	−.21*** [−0.31, −0.12]	−.13** [−0.23, −0.03]	.19*** [0.09, 0.28]			40.78	13.83
(5) Gender	.07 [−0.03, 0.17]	−.25*** [−0.34, −0.16]	−.04 [−0.14, 0.06]	.01 [−0.10, 0.10]		-	-

*Note. n* = 397 for all correlations except those involving age, for which *n* = 387. 95% confidence intervals are shown in square brackets. L-SP = Legitimacy of Secret Power Scale. Gender was coded as 1 = male, 2 = female.

*** *p* < .001. **p < .01.

We tested the associations between the new L-SP scale, participants’ endorsement of masculine honor, and their intentions to cooperate with legal authorities. In line with ICAT, L-SP was positively associated with masculine honor, demonstrating for the first time the existence of an association between the two constructs in the British context. In addition, the more participants legitimized the secret power of criminal groups, the less willing they were to cooperate with legal authorities. These associations offered initial evidence for the convergent validity of the scale. We also observed a negative association between age and L-SP, suggesting that younger people tended to report a more positive view of criminal groups.

## Study 2

In Study 2, we tested L-SP’s measurement model in an independent sample. Additionally, we examined the convergent and divergent validity of the L-SP scale by exploring its associations with theoretically relevant constructs. These constructs included other forms of support for illegality, such as legal cynicism (Sampson & Bartusch, 1998) and support for extrajudicial violence (e.g., Nivette, 2016). Legal cynicism refers to rejecting the norms underlying the laws and a sense that laws need not bind one’s behavior. Support for extrajudicial violence indicates individuals’ endorsement of violence to maintain social order. Differently from the legitimization of criminal groups’ secret power, extrajudicial violence is typically enacted by groups of citizens taking the law into their own hands. We expected L-SP to be positively associated with these two constructs.

We measured participants’ aggressive tendencies (C. D. Barnes et al., 2012) to test whether L-SP merely reflected participants’ aggression. Additionally, we examined two emotional responses to criminal groups, fear of and perceived national threat from those entities (Huddy et al., 2002). Although fear and legitimacy towards authorities might coexist (Jackson et al., 2022), we expected a negative relationship between the two constructs. Moreover, we expected L-SP would be associated with a lower perception that criminal groups threaten the country as a whole.

We also tested participants’ attitudes towards institutions, namely their attitudes towards democracy (Jackson et al., 2013) and the perceived legitimacy of legal authorities (i.e., the police; J. J. Reynolds et al., 2018). We expected L-SP to be associated with more negative views of democracy and the police. A measure of masculine honor was included to test replication of findings from Study 1. Finally, to assess the extent to which participants’ responses to L-SP were linked to a socially desirable response style, we included a measure of social desirability (W. M. Reynolds, 1982). Specifically, we were interested in testing the extent to which individuals who tend to respond in a socially desirable manner were more likely to underreport their positive appraisals of criminal groups’ secret power.

A further objective of Study 2 was to test the practical implications of L-SP by testing a model predicting willingness to cooperate with legal authorities. Cooperation with the police reflects their perceived legitimacy, but it may be inhibited by the fear that criminal groups may arouse in people (see Travaglino & Abrams, 2019). Thus, we tested whether L-SP predicted participants’ willingness to cooperate over and beyond the effects of fear and legitimization of the police.

### Participants and Procedure

Five hundred and one participants from the UK took part in a study on the perception of “illegal social practices” on Prolific via the software Qualtrics (248 male, 247 female, five nonbinary, one did not report their gender). The average age was 45.52 years old (*SD*_age_ = 13.88; see Table D in the Supplemental Material for demographic information). We planned for a sample of *N* = 500. According to a simulation study conducted using the R package pwrSEM (Wang & Rhemtulla, 2021), this sample size provided us with high power (>.99%) to detect the smallest factor loading obtained in Study 1 (.47) for a measurement model with a single latent factor and 20 items, assuming an alpha of .05 and covariances among the residuals of the reversed items. Moreover, a sample of *N* = 500 would enable us to detect small-to-medium coefficients (*ρ* = .12) in a bivariate correlation test (two-tailed) 80% of the time, assuming an alpha of .05 (Faul et al., 2009). After reading a consent form, participants completed the measures. Items within each scale and scale order were randomized. The survey included three attention checks (e.g., “This is an attention check, please select disagree in the scale below”). After completing the survey, participants were debriefed and compensated.

### Measures

The L-SP scale included the 20 items retained from Study 1 (1 = *strongly disagree*, 7 = *strongly agree*). Participants’ endorsement of masculine honor was measured using the same measure as in Study 1 (α = .90). A full list of items used in this study is available at the OSF (https://osf.io/v895f/).

#### Attitudes towards democracy

Attitudes toward democracy were measured using two items (Jackson et al., 2013), “Having a democratic system is a good way of governing this country” and “Democracy may have many problems, but it is better than any other system” (1 = *strongly disagree*, 7 = *strongly agree*). The items were correlated (*r* = .83, *p* < .001, 95% CI [0.80, 0.86]) and were averaged.

#### Support for extrajudicial violence

Support for extrajudicial violence was measured using three items. Participants were asked to suppose that a group of neighbors had caught a person “stealing a car,” “frightening the community,” and “harassing a woman” (Nivette, 2016). Participants were then asked about their approval if the group of neighbors beat the person (1 = *strongly disapprove*, 4 = *strongly approve*; α = .93).

#### General aggressive tendencies

Aggressive tendencies were measured using three items employed in previous research (C. D. Barnes et al., 2012), for example, “It doesn’t take much to set me off” (1 = *strongly disagree*, 7 = *strongly agree*; α = .95).

#### Legal cynicism

Participants were asked to rate their agreement with three statements from Sampson and Bartusch’s (1998) Legal Cynicism Scale (e.g., “Laws were made to be broken”; 1 = *strongly disagree*, 5 = *strongly agree*). The items’ reliability was acceptable (α = .66), and they were averaged.

#### Legitimacy of the police

Four items from the Attitudes Towards Police Legitimacy Scale (J. J. Reynolds et al., 2018) were employed to measure participants’ perception of legal authorities (e.g., “Most police officers care about the communities they work in”; 1 = *strongly disagree*, 5 = *strongly agree*; α = .83).

#### National threat of criminal groups

The perceived national threat of criminal groups was measured using three items (e.g., “To what extent do you think that the presence of criminal groups is a concern in the UK?”; 1 = *not at all*, 7 = *a great deal*; α = .90). Criminal groups were defined to participants as “mafias, cartels, gangs, vigilantes, and other similar organizations.”

#### Fear of criminal groups

A single item was used to measure participants’ fear of criminal groups. Participants read,

Please take a moment to think about when you read or hear about criminal groups in national, local, or international news. This may include reports on mafias, cartels, gangs, vigilantes, or other similar organizations that operate outside the bounds of the law. As you reflect on these news stories, consider what you experience.

followed by the item, “When I read or hear about criminal groups in the news, I feel fearful” (1 = *not at all*, 7 = *a great deal*).

#### Willingness to cooperate with legal authorities

Willingness to cooperate was measured using three items (e.g., “If the situation arose, how likely would you be to report to the police suspicious activity by criminal groups near your house?”; 1 = *very unlikely*, 5 = *very likely*; α = .94). Participants were provided with the same definition of criminal groups employed for the measure of national threat.

#### Social Desirability Scale

Socially desirable tendencies were measured using the Marlowe–Crowne Social Desirability Scale Short Form (W. M. Reynolds, 1982). The scale includes 13 statements (1 = *true*, 2 = *false*; e.g., “I have never deliberately said something that hurt someone’s feelings”; α = .74). Higher scores indicate a stronger tendency to respond in a socially desirable manner.

#### Demographic variables

Participants provided information about their age, gender, ethnicity, level of education, student and employment status, region of provenience, subjective social status, and political orientation (1 = *I am a left-winger*, 10 = *I am a right-winger*). Subjective social status was measured by asking participants to select their position on a ladder from 1 (*lowest*) to 10 (*highest*) after reading a brief set of instructions explaining that the top of the ladder indicated people who are “best off” (most money, education, and best jobs) and the bottom, people who are “worst off” (least money, education, and worst or no jobs; Adler et al., 2000).

### Results and Discussion

Means and standard deviations for the variables are summarized in Table 3. Fourteen participants failed an attention check and were excluded from the subsequent analyses. The final sample was *N* = 487.

**Table 3.** Means and standard deviations for variables: Study 2.

Variable	*M*	*SD*
L-SP scale	2.31	0.96
Support for extrajudicial violence	2.37	1.17
Honor ideology for masculinity	3.26	1.27
Attitudes towards democracy	4.15	0.88
Willingness to cooperate with legal authorities	3.59	1.14
General aggressive tendencies	2.83	1.43
Legal cynicism	2.05	0.83
Legitimacy of the police	3.38	0.85
National threat of criminal groups	4.77	1.28
Fear	3.73	1.69
Education	6.06	1.45
Subjective social status	5.78	1.45
Political orientation	4.52	2.09
Age	45.54	13.87

*Note.* Education was coded from 1 (some primary education) to 8 (graduate or professional degree); Political orientation was coded from 1 (I am a left-winger) to 10 (I am a right-winger). Subjective social status was coded from 1 (lowest) to 10 (highest). L-SP = Legitimacy of Secret Power Scale.

#### Testing the measurement model of the L-SP scale

To confirm the measurement model of L-SP, we employed a confirmatory factor analysis, with maximum likelihood as the extraction method and robust standard errors to accommodate deviations from normality assumptions. We estimated a one-factor measurement model, which yielded an excellent fit (CFI = .95, RMSEA = .06, 90% CI [0.05, 0.07], SRMR = .04), confirming L-SP’s factor structure in an independent sample.^1^ The factor loadings for the models are summarized in Tables E–F, Supplemental Material. The scale exhibited robust internal reliability (α = .95); values of skewness (0.64) indicated a pronounced departure from the normal distribution; excess kurtosis (−0.16) was minimal.

#### Testing the relationships between L-SP and other constructs

Correlations between L-SP, social desirability, and demographics are summarized in Table 4. The correlation between L-SP and social desirability was significant but small. This finding indicates that participants’ responses to L-SP were only minimally related to a socially desirable response style. Among the demographic variables, only age had a more substantial association with L-SP, suggesting that younger people tend to legitimize the secret power of criminal groups more strongly than older people.

**Table 4.** Correlations of the Social Desirability Scale and demographic variables with L-SP.

Variable	*r*	95% CI	*df*	*p* value
Social desirability	−.13	[−0.22, −0.04]	485	.004
Gender	−.02	[−0.11, 0.07]	479	.694
Age	−.26	[−0.34, −0.17]	485	< .001
Education	.01	[−0.09, 0.09]	481	.962
Employment	−.06	[−0.15, 0.03]	476	.205
Subjective social status	.10	[0.01, 0.19]	485	.028
Political orientation	−.02	[−0.11, 0.07]	485	.622

*Note. df* = degrees of freedom. Gender: 1 = male, 2 female; employment: 1 = employed, 2 = unemployed. Education was coded from 1 (some primary education) to 8 (graduate or professional degree); political orientation was coded from 1 (I am a left-winger) to 10 (I am a right-winger); subjective social status was coded from 1 (lowest) to 10 (highest). L-SP = Legitimacy of Secret Power Scale.

**Figure 1.**
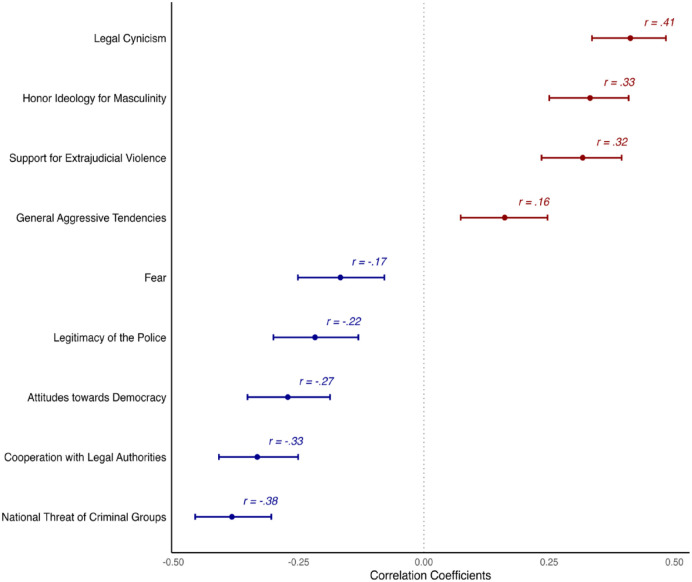
Correlation of the Legitimacy of Secret Power Scale and other variables: Study 2. *Note.* Bars represent the 95% confidence interval of the correlation coefficients. All correlations were significant at *p* < .001.

Figure 1 displays the correlation coefficients and 95% confidence intervals for the relationships between the L-SP scale and the other constructs (correlations among all variables are summarized in Table G, Supplemental Material). The L-SP scale was positively correlated with theoretically related constructs of legal cynicism and support for extrajudicial violence. Moreover, L-SP was negatively associated with the perceived national threat of criminal groups and individuals’ willingness to cooperate with legal authorities. The size of these correlations was moderate, providing evidence for the construct’s convergent validity.

The correlation between L-SP and individuals’ general aggressive tendencies was positive but small, indicating that individuals’ aggressive tendencies were only very weakly associated with the legitimization of secret power. This suggests that support for criminal groups’ governance activities, as measured by the L-SP scale, does not map onto individuals’ own propensity for aggression.

Notably, we expected a stronger correlation between L-SP and fear, but this association was small. Fear is typically shaped by the direct threat to one’s immediate environment (Ferraro, 1995; Huddy et al., 2002). Thus, this result could imply that people view the legitimacy of criminal groups in relation to their broader implications for social order and governance. This finding is also consistent with research indicating that fear and legitimacy toward authorities might coexist in some circumstances (Jackson et al., 2022).

Finally, the L-SP scale was associated with masculine honor and attitudes towards democracy. In line with prior studies, participants who endorsed masculine honor more strongly were also more likely to legitimize criminal groups. Participants who tended to accept the power of criminal groups also reported lower support for democracy. Democracy is predicated on the rule of law, transparency, and participation. Accepting criminal groups’ power may reflect disillusionment with these ideals.

#### Predicting willingness to cooperate with legal authorities

We tested L-SP’s role in a model to predict individuals’ willingness to cooperate with legal authorities. We employed structural equation models with latent variables and maximum likelihood with robust standard errors. Specifically, we tested a model in which the L-SP scale, the perceived legitimacy of the police, and fear of criminal groups predicted individuals’ willingness to cooperate. Participants’ age, gender, employment, education, subjective social status, and political orientation were included as covariates in the model to control for their effects. Repeating the analyses without covariates led to the same conclusions.

**Figure 2.**
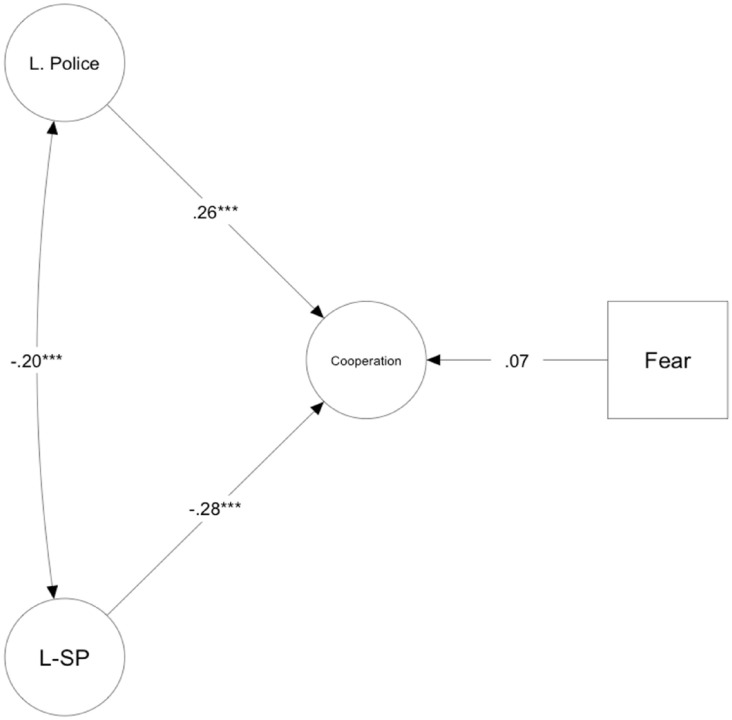
Structural model with standardized coefficients predicting participants’ willingness to report criminal group activities. *Note.* Age, gender (1 = male, 2 = female), subjective social status, employment (1 = employed, 2 = unemployed), education, and political orientation are covariates in the model. Coefficients are standardized. The latent variables’ measurement model is omitted for clarity. L. police = Legitimacy of the Police; L-SP = Legitimacy of Secret Power Scale; Cooperation = Willingness to Cooperate with Legal Authorities. ***p < .001.

Figure 2 displays the model’s results, and Table 5 summarizes the model’s structural parameters. The model fit was adequate, CFI = .94, RMSEA = .05, 90% CI [0.04, 0.05], SRMR = .06. After accounting for the effects of fear, perceived police legitimacy, and the covariates, the L-SP scale was negatively associated with willingness to cooperate. Thus, L-SP explained variance in cooperation over and beyond the effects of the other constructs.

**Table 5.** Model parameters predicting willingness to cooperate with legal authorities.

Predictors	β	*SE*	*z*	*p*	95% CI
L-SP	−.29	0.05	−5.80	< .001	[−0.38, −0.19]
Legitimacy of the police	.26	0.07	4.68	< .001	[0.15, 0.36]
Fear	.07	0.03	1.54	.124	[−0.03, 0.16]
Age	.06	0.01	1.33	.185	[−0.03, 0.15]
Gender	−.02	0.10	−0.34	.735	[−0.10, 0.07]
Employment	.03	0.12	0.51	.609	[−0.07, 0.12]
Education	.11	0.04	2.20	.028	[0.01, 0.20]
Subjective social status	−.12	0.04	−2.52	.012	[−0.22, −0.03]
Political orientation	−.01	0.02	0.17	.866	[−0.08, 0.09]

*Note.* Coefficients are standardized. The criterion variable in the analyses was willingness to report criminal group activity to the police. Gender: 1 = male, 2 = female; employment: 1 = employed, 2 = unemployed. L-SP = Legitimacy of Secret Power Scale.

## Study 3

Study 3 had three objectives. First, we aimed to confirm the measurement model of the L-SP scale in different contexts and languages and test for its measurement stability. Measurement stability (or invariance) is essential for comparing a construct across contexts because it indicates that the items are interpreted in similar ways (Fischer & Karl, 2019; Van de Vijver & Leung, 2021).

In the current study, we sampled participants from Italy, the UK, Japan, and the US. These countries are all affected by instances of secret power, although its expressions vary. In Italy, criminal organizations have long exerted control over territories and communities, forming parallel systems of governance (Travaglino & Abrams, 2019). In the United Kingdom, large gangs are known to undertake some of the core functions of the state, controlling neighborhoods and territories (Campana & Varese, 2018; Densley, 2013). In Japan, albeit weakened by state interventions, the yakuza seeks visibility to maintain influence over communities (Baradel & Bortolussi, 2021). In the United States, gangs and criminal organizations create subcultures that exert social control (Akerlof & Yellen, 1993; Sánchez-Jankowski, 2018). Differences in the prevalence of criminal groups within countries, the distinct relationships those groups can establish with communities, and the varying configuration of state and secret power provide an ample spectrum to examine the performance of the L-SP scale.

Second, we examined the associations between L-SP and other theoretically relevant constructs in each country. In addition to testing the associations between L-SP and most of the constructs examined in Study 2, we included a measure of anger against criminal groups because of the importance of this emotion in people’s reactions and opposition to criminal activities (D. Johnson, 2009). Instead of support for extrajudicial violence, we included a more general measure of participants’ civic honesty in order to examine the cross-contextual relationship between L-SP and acceptance of corruption. Further, we included a measure of SDO to explore the role of a different ideology in participants’ appraisals of secret power.

Finally, we tested the model predicting participants’ willingness to cooperate with legal authorities across countries. As in Study 2, we tested an application of L-SP by examining whether and in which settings it could predict participants’ cooperation while controlling for the perceived legitimacy of the police and fear of criminal groups.

### Participants and Procedure

An initial sample of 2,502 participants was contacted to take part in a study on the “perceptions of social practices and groups.” Data were collected via Qualtrics by a specialized global market research firm. Participants could take part if they were residents of the US, the UK, Japan, or Italy. After removing nonresident participants, the final sample was of 2,389: *n*_US_ = 604 (*M*_age_ = 44.93, *SDage* = 14.41; 328 women); *n*_UK_ = 582 (*M*_age_ = 44.08, *SDage* = 13.98; 303 women); *n*_IT_ = 600 (*M*_age_ = 45.58, *SDage* = 13.72; 301 women); *n*_JA_ = 603 (*M*_age_ = 46.38, *SDage* = 13.68; 298 women). Samples were representative in terms of macroregion of provenience in the respective countries. Demographic characteristics of the samples are summarized in Tables H–K, in the Supplemental Material.

We planned for a sample of at least *N =* 500 per country because this would afford us sufficient statistical power (> .99%) to detect the smallest factor loading obtained in Study 1(.47) in each country for a measurement model with a single latent factor and 20 items, assuming an alpha of .05 (Wang & Rhemtulla, 2021). The sample would also allow us to detect small-to-medium coefficients (*ρ* = .12) in a bivariate correlation test (two-tailed) 80% of the time, assuming an alpha of .05 (Faul et al., 2009). Due to our limited control over the data collection process, we decided to oversample and recruit 600 participants per country, a precautionary measure designed to compensate for potential exclusions.

Materials were prepared in English and translated into Italian and Japanese. The measures, including the new L-SP scale, were back-translated following guidelines from Brislin (1986). Participants first completed the L-SP scale. The order of subsequent scales was randomized. The presentation of items within all scales was also randomized. The survey included an attention check, “This is an attention check, please select disagree in the scale below.” After completing the survey, participants were debriefed and compensated for their time.

### Measures

Participants completed the L-SP scale (20 items) and measures of legal cynicism (three items; α = .63), perceived national threat (three items; α = .89), willingness to report criminal group activities to legal authorities (three items; α = .91), fear of criminal groups (one item), and attitudes towards democracy (two items; *r* = .75, *p* < .001, 95% CI [0.73, 0.77]) as described in Study 2. In this study, we measured participants’ legitimization of the police employing the full 11-item scale (α = .93), and masculine honor using eight items (α = .87). Participants also completed three additional measures.

#### Civic honesty

Civic honesty was measured using four items from the Morally Debatable Behaviors Scale (Harding et al., 1986) included in the World Values Survey (Haerpfer et al., 2022). Participants were asked whether the following behaviors were justifiable, “Claiming state benefits to which you are not entitled,” “Avoiding a fare on public transport,” “Cheating on taxes if you have a chance,” “Someone accepting a bribe in the course of their duties” (1 = *never justifiable*, 10 = *always justifiable*). Items were reverse-coded and averaged (α = .87).

#### Anger against criminal groups

Anger against criminal groups was measured by employing a single item, “When I read or hear about criminal groups in the news, I feel angry” (1 = *not at all*, 7 = *a great deal*). The item followed the same instructions employed to measure fear.

#### Social dominance orientation

SDO was measured using four items (Aichholzer & Lechner, 2021). A sample item is “Superior societal groups should dominate inferior groups” (1 = *strongly disagree*, 7 = *strongly agree*; α = .69).

#### Demographic variables

Participants completed measures of gender, age, employment status, student status, income, ethnicity (residence in Japan), and region of residence. Education level and income had a different number of categories across countries and were transformed by assigning participants a percentile rank based on the distributions in each country. This method standardizes responses, allowing for comparison despite the differences in the number of response categories.

### Results and Discussion

Means and standard deviations for the variables in the study are summarized in Table 6. Thirty-five participants failed the attention checks in the UK, 14 in the US, 20 in Italy, and 31 in Japan. These participants were excluded from the analyses. The remaining sample was *N* = 2,289 (*n*_US_ = 590, *n*_UK_ = 547, *n*_IT_ = 580, *n*_JA_ = 572).

**Table 6.** Means and standard deviations across countries: Study 3.

Variables	*M* (*SD*)			
	UK	US	Italy	Japan
**Study variables**				
L-SP	2.50 (1.09)	2.68 (1.26)	1.97 (0.95)	2.20 (0.94)
Legal cynicism	2.38 (0.92)	2.37 (1.04)	2.40 (0.83)	1.92 (0.77)
SDO	2.84 (1.06)	2.83 (1.19)	2.34 (1.08)	3.46 (0.83)
Masculine honor	3.67 (1.31)	4.17 (1.39)	3.66 (1.13)	3.88 (1.04)
Fear	4.16 (1.56)	4.16 (1.81)	4.92 (1.63)	4.80 (1.44)
Legitimacy of the police	3.38 (0.82)	3.62 (0.92)	3.70 (0.66)	3.44 (0.73)
Willingness to cooperate	3.57 (1.15)	3.97 (1.06)	4.04 (1.01)	3.59 (1.05)
Attitudes towards democracy	3.79 (0.90)	3.96 (1.03)	4.14 (0.81)	3.76 (0.79)
Anger	4.78 (1.58)	4.77 (1.78)	5.86 (1.37)	4.86 (1.48)
National threat crim. groups	5.01 (1.30)	5.33 (1.33)	6.12 (1.04)	4.87 (1.22)
Civic honesty	8.33 (1.89)	7.97 (2.34)	8.74 (1.71)	9.02 (1.41)
**Demographics**				
Income	2.34 (1.08)	3.07 (1.80)	1.79 (0.76)	3.13 (1.57)
Education	5.30 (1.51)	3.87 (1.56)	5.35 (1.80)	7.52 (1.65)
SES	6.28 (1.76)	5.54 (2.03)	5.44 (1.53)	6.28 (1.81)
Political orientation	5.22 (2.06)	6.05 (2.87)	5.56 (2.62)	5.83 (1.45)
Age	44.52 (13.99)	45.13 (14.38)	45.54 (13.83)	46.55 (13.58)

*Note.* L-SP = Legitimacy of Secret Power Scale; SDO = Social Dominance Orientation; Willingness to Cooperate = Willingness to Cooperate with Legal Authorities; SES = Subjective Socioeconomic Status. Political orientation was coded from 1 (I am a left-winger) to 10 (I am a right-winger); Subjective Social Status was coded from 1 (lowest) to 10 (highest). The categories employed in the variable Education in each country are described in the Supplemental Material, Tables H–K.

#### Testing the measurement model and stability of the L-SP scale

We conducted confirmatory factor analyses to confirm the structure of the L-SP scale. In each country, we specified a single-factor model, using maximum likelihood with robust standard errors as extraction method. The models had an excellent fit across countries (see Table 7). The standardized factor loadings for the models are summarized in Table 8. Although in Japan two items had relatively lower loadings (L-SP21_R and L-SP34_R), the fit indices confirmed the L-SP scale’s structure across four different countries.

**Table 7.** Measurement model fit indices across countries: Study 3.

Country	CFI	RMSEA	SRMR
United Kingdom	.98	.04 [0.03, 0.05]	.03
United States	.97	.05 [0.04, 0.06]	.03
Italy	.97	.05 [0.04, 0.05]	.03
Japan	.98	.04 [0.03, 0.05]	.03

*Note.* RMSEA 90% confidence intervals are displayed in square brackets.

**Table 8.** Standardized factor loadings of the L-SP scale across countries: Study 3.

Items	λ			
	UK	US	Italy	Japan
L-SP1	.81	.82	.69	.71
L-SP3	.80	.80	.77	.79
L-SP2	.80	.84	.72	.73
L-SP27	.79	.78	.86	.74
L-SP19	.79	.78	.75	.78
L-SP4	.78	.83	.67	.69
L-SP28	.77	.81	.79	.79
L-SP5	.76	.80	.83	.73
L-SP32	.75	.79	.78	.80
L-SP17	.75	.79	.75	.81
L-SP7	.74	.72	.74	.76
L-SP38	.70	.71	.71	.72
L-SP30	.69	.75	.59	.77
L-SP40	.69	.78	.73	.66
L-SP24	.65	.68	.63	.57
L-SP10_R	.56	.52	.40	.49
L-SP22_R	.54	.46	.42	.57
L-SP21_R	.52	.49	.43	.22
L-SP13_R	.40	.43	.38	.29
L-SP34_R	.39	.50	.37	.16

*Note*. L-SP = Legitimacy of Secret Power Scale.

We proceeded to test the measurement stability (or invariance) of the L-SP scale. We first specified L-SP’s measurement model, examining whether this was the same across countries (configural invariance). We then proceeded to test whether factor loadings could be constrained to be the same across countries without significantly worsening the model’s fit (metric invariance). Finally, we constrained intercepts to be the same (scalar invariance). Analyses were conducted using a multigroup structural equation model. To assess deterioration in the model fit, we considered the ΔCFI (⩽ .01), ΔGamma hat (⩽ .001), and ΔMcDonald’s noncentrality index (NCI; ⩽.02) criteria (Chen, 2007; Cheung & Rensvold, 2002).

L-SP had excellent configural fit, CFI = .97, RMSEA = .04, 90% CI [0.04, 0.05], SRMR = .03. Constraining the loadings to be the same across groups did not significantly deteriorate the model’s fit according to two of the three criteria, ΔCFI = −.003, ΔMcDonald’s NCI = −.02; the ΔGamma hat = −.003 indicated a slight discrepancy. Overall, these results were compatible with a fully metric invariant model. Constraining the intercepts to be equal across countries resulted in a model misfit higher than the thresholds across all indices, ΔCFI = −.017, ΔMcDonald’s NCI = −.071, ΔGamma hat = −.013. We, therefore, tested a partially invariant model after identifying which of the intercepts were causing the strongest misfit and allowing them to vary freely across countries (Fischer & Karl, 2019). By releasing the constraints on the intercepts of nine items (Items 30, 34, 21, 4, 19, 22, 7, 17, and 13; see Table 1), we achieved partial scalar invariance according to ΔCFI = −.005 and ΔMcDonald’s NCI = −.020. The ΔGamma hat = −.004 indicated a slight discrepancy. Partial scalar invariance is typically a more realistic goal in cross-country research (Steinmetz, 2018). Thus, these analyses supported the full metric and partial scalar stability of the L-SP scale across different languages and countries.

#### Testing the L-SP scale’s relationships with other constructs

**Figure 3.**
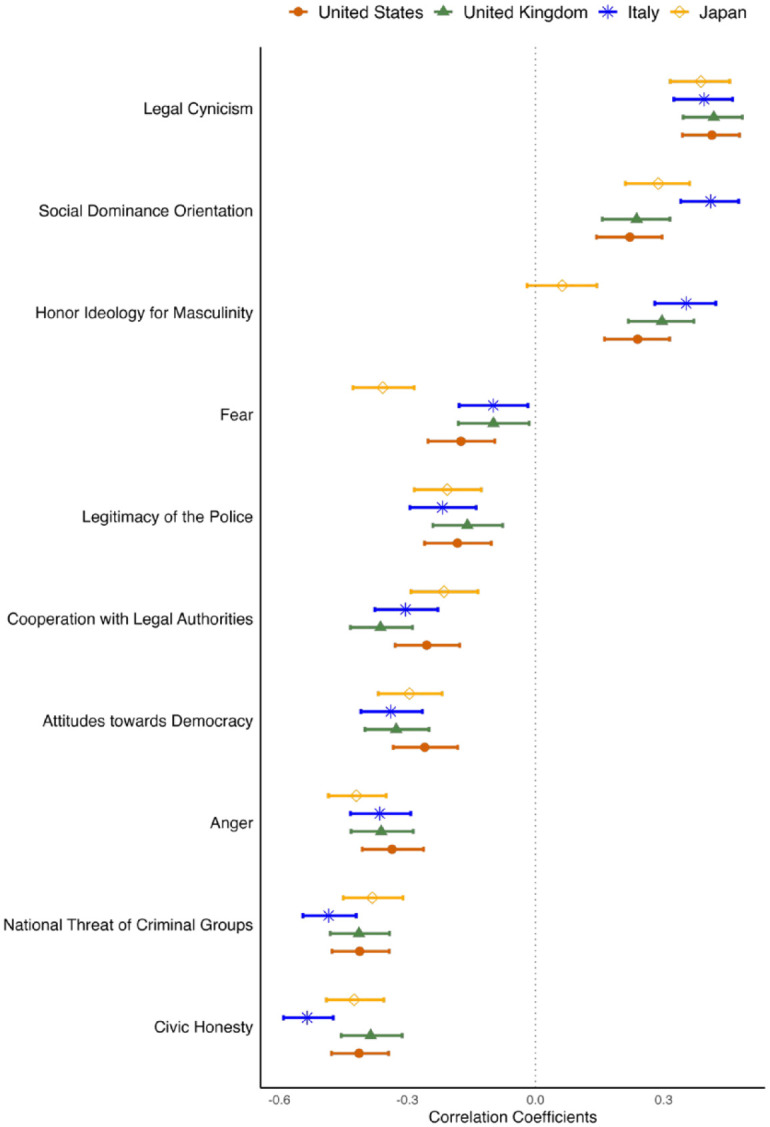
Correlations between L-SP and other variables across countries: Study 3. *Note.* Bars represent 95% confidence intervals of the correlation coefficients.

Correlations between the L-SP scale and other constructs are summarized in Table 9 and displayed in Figure 3. Correlations between L-SP and demographic variables are displayed in Table 10 (see Tables N–Q in the Supplemental Material for correlations among all constructs). The distribution of the L-SP scale departed from normality in each country (skew_US_ = 0.42, skew_UK_ = 0.38, skew_IT_ = 1.37, skew_JA_ = 0.59; excess kurtosis_US_ = −0.82, kurtosis_UK_ = −0.83, kurtosis_IT_ = 1.99, kurtosis_JA_ = −0.73). Departures were especially pronounced in the Italian sample. The relationships between L-SP and the other constructs were mostly consistent across countries, supporting the scale’s convergent validity in all settings. Specifically, participants who tended to legitimize secret power were more prone to legal cynicism and less likely to endorse standards of civic honesty.

**Table 9.** Correlations between L-SP and demographic variables: Study 3.

	**United Kingdom**				**United States**			
Variable	*r*	95% CI	*df*	*p*	*r*	95% CI	*df*	*p*
Gender	.08	[−0.003, 0.16]	538	.059	.04	[−0.04, 0.12]	587	.303
Income	.01	[−0.09, 0.09]	508	.992	−.05	[−0.13, 0.03]	576	.219
Education	−.04	[−0.13, 0.04]	538	.305	−.06	[−0.14, 0.02]	588	.150
Employment	−.05	[−0.13, 0.04]	545	.274	−.07	[−0.15, 0.01]	588	.093
SES	.05	[−0.03, 0.14]	545	.205	−.08	[−0.15, 0.01]	588	.069
Political orientation	−.03	[−0.11, 0.05]	545	.488	.08	[0.0008, 0.16]	578	.048
Age	−.31	[−0.38, −0.23]	545	< .001	−.35	[−0.42, −0.28]	588	< .001
	**Italy**				**Japan**			
	*r*	95% CI	*df*	*p*	*r*	95% CI	*df*	*p*
Gender	.09	[−0.17, −0.01]	575	.036	−.01	[−0.08, 0.08]	567	.981
Income	−.07	[−0.15, 0.02]	519	.138	−.07	[−0.15, 0.02]	486	.144
Education	−.06	[−0.14, 0.02]	574	.127	−.04	[−0.12, 0.04]	568	.308
Employment	−.03	[−0.11, 0.05]	578	.440	−.04	[−0.12, 0.04]	570	.357
SES	−.06	[−0.14, 0.02]	578	.130	.01	[−0.07, 0.09]	570	.865
Political orientation	.15	[0.06, 0.22]	578	< .001	.04	[−0.04, 0.12]	570	.318
Age	−.21	[−0.28, −0.13]	578	< .001	−.25	[−0.32, −0.17]	570	< .001

*Note.* Gender: 1 = male, 2 = female; Income and Education were percentile ranks (higher values indicate more education and higher income). Employment: 1 = employed, 2 = unemployed. Political orientation was coded from 1 (I am a left-winger) to 10 (I am a right-winger). SES = Subjective Socioeconomic Status; L-SP = Legitimacy of Secret Power Scale.

**Table 10.** Correlations between L-SP scale and the other variables: Study 3.

	**United Kingdom**				**United States**			
Variable	*r*	95% CI	*df*	*p*	*r*	95% CI	*df*	*p*
Masculine honor	.30	[0.22, 0.37]	545	< .001	.24	[0.16, 0.31]	588	< .001
Attitudes toward democracy	−.33	[−0.40, −0.25]	545	< .001	−.26	[−0.33, −0.18]	588	< .001
Cooperation	−.36	[−0.43, −0.29]	545	< .001	−.26	[−0.33, −0.18]	588	< .001
Legal cynicism	.42	[0.35, 0.48]	545	< .001	.41	[0.34, 0.48]	588	< .001
Legitimacy of the police	−.16	[−0.24, −0.08]	545	< .001	−.18	[−0.26, −0.10]	588	< .001
National threat	−.41	[−0.48, −0.34]	545	< .001	−.41	[−0.48, −0.34]	588	< .001
Fear	−.10	[−0.18, −0.01]	545	.021	−.18	[−0.25, −0.10]	588	< .001
Anger	−.36	[−0.43, −0.29]	545	< .001	−.34	[−0.41, −0.26]	588	< .001
Civic honesty	−.39	[−0.46, −0.31]	545	< .001	−.41	[−0.48, −0.34]	588	< .001
Social dominance orientation	.24	[0.16, 0.31]	545	< .001	.22	[0.14, 0.30]	588	< .001
	**Italy**				**Japan**			
	*r*	95% CI	*df*	*p*	*r*	95% CI	*df*	*p*
Masculine honor	.35	[0.28, 0.42]	578	< .001	.06	[−0.02, 0.14]	570	.137
Attitudes toward democracy	−.34	[−0.41, −0.26]	578	< .001	−.30	[−0.37, −0.22]	570	< .001
Cooperation	−.30	[−0.38, −0.23]	578	< .001	−.22	[−0.29, −0.14]	570	< .001
Legal cynicism	.40	[0.32, 0.46]	578	< .001	.39	[0.32, 0.45]	570	< .001
Legitimacy of the police	−.22	[−0.29, −0.14]	578	< .001	−.21	[−0.28, −0.13]	570	< .001
National threat	−.49	[−0.54, −0.42]	578	< .001	−.38	[−0.45, −0.31]	570	< .001
Fear	−.10	[−0.18, −0.02]	578	.017	−.36	[−0.43, −0.28]	570	< .001
Anger	−.37	[−0.43, −0.29]	578	< .001	−.42	[−0.49, −0.35]	570	< .001
Civic honesty	−.54	[−0.59, −0.47]	578	< .001	−.43	[−0.49, −0.36]	570	< .001
Social dominance orientation	.41	[0.34, 0.48]	578	< .001	.29	[0.21, 0.36]	570	< .001

*Note.* Cooperation = Willingness to Cooperate with Legal Authorities; L-SP = Legitimacy of Secret Power Scale.

Moreover, L-SP was negatively correlated with emotional responses to criminal groups, including national threats, fear, and anger. The correlations between L-SP and fear were small in all countries except Japan, where its magnitude was moderate. We explored whether in Japan this correlation coefficient differed from that of the relationship between L-SP and national threat (cf. Huddy et al., 2002), employing Zou’s (2007) confidence interval for contrasting dependent correlations with overlapping variables. We found that in Japan the association between L-SP and fear was not significantly different from that of L-SP and national threat, Δ*r*_JA_ = .02, 95% CI_JA_ [−0.06, 0.10] (these coefficients were significantly different in all other samples, Δ*r* ⩾ .24). This pattern suggests the existence of a stronger overlap between national threat and personal fear in Japan.

There were also negative associations between L-SP and participants’ attitudes towards democracy and the perceived legitimacy of the police. Moreover, L-SP was positively associated with participants’ SDO and, except in Japan, masculine honor. Zou’s confidence interval test confirmed that whereas the size of the relationships between L-SP and SDO and L-SP and masculine honor were not significantly different in each of the countries (Δ*r* ⩽ −.02), they significantly differed in Japan (Δ*r*_JA_ = .23, 95% CI_JA_ [−0.02, 0.14]).

#### Predicting willingness to cooperate with legal authorities

We tested a model predicting participants’ willingness to cooperate with legal authorities across countries using a multigroup structural equation model with latent variables. Predictors in the model were L-SP, legitimization of the police, and fear.^2^ We also controlled for demographic characteristics of age, gender, employment, income, subjective social status, and political orientation. Repeating the analyses without covariates led to the same conclusions. The model’s fit was adequate, CFI = .94, RMSEA = .04, 90% CI [039, .043], SRMR = .08.

To test cross-country differences in the magnitude of the relationships, we compared the unconstrained model to one where all structural paths of the focal variables were constrained to be equal across groups. Model comparisons were conducted using a scaled chi-squared difference test (Satorra & Bentler, 2001). The fully constrained model had a significantly worse fit, Δχ^2^_(*df* = 9)_ = 23.29, *p* = .006, indicating that the magnitude of some of the paths changed among countries. We proceeded by systematically constraining each of the structural paths. Constraining the paths of L-SP, Δχ^2^_(*df* = 3)_ = 13.15, *p* = .004, and perceived legitimacy of the police, Δχ^2^_(*df* = 3)_ = 8.05, *p* = .045, resulted in a significantly worse model fit. Constraining fear did not result in a significantly worse fit, Δχ^2^_(*df* = 3)_ = 3.25, *p* = .324. Thus, we interpreted a model in which only the association between fear and participants’ willingness to cooperate was constrained to be equal across countries.

The model’s focal parameters are summarized in Table 11 (the effects of the covariates are summarized in Table R, Supplemental Material). Standardized coefficients can be used to compare the relative weight of the parameter within the sample, whereas unstandardized coefficients can be interpreted in cross-country comparisons (Kline, 2016). L-SP significantly predicted participants’ willingness to cooperate with legal authorities in all contexts. Notably, the magnitude of the path from L-SP to participants’ willingness to cooperate was not significantly different from the magnitude of the path of perceived legitimacy of the police on willingness to cooperate, Δ*b*_UK_ = −0.07, 95% CI_UK_ [−0.23, 0.08]; Δ*b*_IT_ = 0.01, 95% CI_IT_ [−0.14, 0.15]; Δ*b*_JA_ = −0.06, 95% CI_JA_ [−0.20, 0.09], except in the U.S. sample, Δ*b*_US_ = −0.27, 95% CI_US_ [−0.42, −0.13]. Overall, results emphasize the importance of considering participants’ appraisal of secret power in their intentions to cooperate with legal authorities.

**Table 11.** Parameters for the model predicting willingness to report criminal groups’ activity across countries: Study 3.

Predictors	β	*b*	*SE*	*z*	*p*	95% CI
**United Kingdom**						
Legitimacy of Secret Power (L-SP)	−.29	−0.25	0.04	−6.29	< .001	[−0.37, −0.20]
Legitimacy of the police	.36	0.47	0.07	6.82	< .001	[0.26, 0.46]
Fear	.11	0.07	0.02	4.71	< .001	[0.06, 0.15]
**United States**						
Legitimacy of Secret Power (L-SP)	−.16	−0.10	0.03	−3.91	< .001	[−0.24, −0.08]
Legitimacy of the police	.43	0.45	0.06	8.20	< .001	[0.33, 0.53]
Fear	.145	0.07	0.02	4.71	< .001	[0.08, 0.19]
**Italy**						
Legitimacy of Secret Power (L-SP)	−.24	−0.20	0.04	−5.38	< .001	[−0.32, −0.15]
Legitimacy of the police	.23	0.33	0.07	4.55	< .001	[0.13, 0.32]
Fear	.13	0.07	0.02	4.71	< .001	[0.07, 0.18]
**Japan**						
Legitimacy of Secret Power (L-SP)	−.12	−0.13	0.04	−2.77	.006	[−0.21, −0.03]
Legitimacy of the police	.18	0.25	0.07	3.60	< .001	[0.08, 0.27]
Fear	.10	0.07	0.02	4.71	< .001	[0.06, 0.15]

## General Discussion

When citizens’ relationship with institutions deteriorates, and legal bodies suffer from a decline in legitimacy (Tyler, 2023), it becomes increasingly urgent to address how different forms of power and authority operate. In the present research, we laid the foundations for investigating the psychology of criminal authority by developing and validating a robust measure of the legitimization of criminal groups’ secret power. In three studies, we explored and confirmed the measurement model for a new Legitimacy of Secret Power (L-SP) Scale. The scale tapped into individuals’ appraisal of a broad range of governance functions exerted by criminal groups. The scale had a consistent structure across studies. Moreover, it exhibited robust measurement stability across four distinct national contexts—the UK, the US, Italy, and Japan—demonstrating its suitability for comparative research.

We tested the associations of the L-SP scale with other theoretically relevant constructs. Participants who perceived secret power as more legitimate also endorsed other forms of illegality—as shown by associations with stronger legal cynicism, support for extrajudicial violence, and diminished honesty in civic contexts. They held more negative views of the institutional authority, as indicated by correlations with lower perceived legitimacy of the police and more negative attitudes towards democracy. Additionally, these individuals exhibited a reduced negative emotional response, as indicated by a diminished sense of national threat and lower levels of anger and, to a lesser extent, fear toward criminal groups.

Study 3 showed that the associations were broadly consistent across the countries included in the research. There were, however, two notable exceptions. In Japan, the relationship between L-SP and fear was similar in magnitude to that of L-SP and perceived national threat. In the other countries, L-SP’s association with national threat was significantly stronger than that of L-SP and personal fear. This result could reflect specific cultural, social, or historical influences in Japan that shape a different relationship between perceptions of criminal authority and emotional responses of personal fear to it. Furthermore, while L-SP’s associations with masculine honor and SDO were similar in magnitude across all countries, the association between L-SP and SDO was significantly stronger in Japan compared to that of L-SP and masculine honor. From the perspective of intracultural appropriation theory, this result could reflect the different ideological instances appropriated by criminal forms of authority in the Japanese context, which could emphasize less male aggression and more hierarchical structures and status dominance (Zhang et al., 2005).

Finally, across studies, we tested the role of the perceived legitimacy of secret power in predicting participants’ willingness to cooperate with legal authorities. L-SP was significantly associated with cooperation when controlling for the effects of the perceived fear of criminal groups and the perceived legitimacy of legal authorities. Interestingly, Study 3 showed that across all countries, except the United States, the association between L-SP and cooperation was equivalent in magnitude to that of the perceived legitimacy of legal authorities and cooperation. This finding demonstrates the scale’s utility in explaining a phenomenon of strong practical relevance. Cross-country differences underscore the importance of investigating the dynamics of criminal authority across different areas and in diverse groups of participants.

### Theoretical Implications, Limitations, and Future Directions

The study of secret power extends our understanding of power dynamics and legitimacy by considering a wider array of entities simultaneously shaping social order and governance. The concept of secret power encompasses a broad range of informal political relations that occur in parallel to, in competition, and sometimes in cooperation with legal institutions. These relations involve systems that, although criminal, may be highly structured and regulated, and whose reach might extend to people who do not behave illegally themselves. As such, the notion of secret power does not coincide with the conventional notion of immoral or tyrannical authority (e.g., Haslam & Reicher, 2007). Rather, it stresses how people often must navigate complex webs of overlapping and informal power systems beyond state authority.

Consistent with this focus, our findings indicate that the legitimization of secret power operates on dimensions that are distinct from the conventional left–right political spectrum. The weak associations between L-SP and participants’ political orientation across studies indicate that the appraisal of secret power is not a direct reflection of this spectrum. Instead, in line with ICAT, secret power aligns more closely with alternative ideological frameworks and values, such as masculine honor and considerations about hierarchies. Criminal groups can gain stronger legitimacy as long as their actions align with standards considered important and accepted in a given context. This can make even their use of violence more tolerable (cf. Fiske & Rai, 2015; Jackman, 2001).

Legitimization of secret power is also unlikely to reflect two of the needs postulated by general theories of system justification, those for security and reduced uncertainty (Jost, 2019). This is because the groups exerting secret power are criminal, unpredictable, and explicitly invalidated by the state while coexisting with it. Criminal systems of power may instead exert essential functions in the eyes of those who may have been left, psychologically or even geographically, at the periphery of formal governance relations. Indeed, the correlations between L-SP and other constructs indicated that the legitimization of secret power is aligned with skepticism towards civic ethical standards and democracy. This pattern of associations suggests that acceptance of secret power might be underpinned by a broader “anti-institutional identity” (Emler & Reicher, 1995). It seems likely that openness to secret power might be elevated in circumstances when trust in legitimate national authority wanes and remains low for protracted periods, or when governments have been exposed by multiple scandals. However, we expect that it is the local conditions that may be more important. The tendency to legitimize secret power may be attenuated if localized, trusted, legitimate authorities exist (cf. Davies et al., 2021). Future research should examine the conditions that precipitate the emergence of such identity and its implications directly.

Some limitations of this research should be acknowledged. Although criminal governance practices are one of its key instantiations, secret power is a highly multifaceted and complex concept. It may encompass different entities operating within or across states, and the scale developed in this research might not have captured all its aspects. Future research might revise and expand the scale to tap into additional domains and areas. For instance, it would be interesting to investigate how secret power operates in the context of other nonstate entities, especially those that are formally legal but exert power without state authorization (e.g., NGOs, churches, global corporations). In addition, research may examine the dynamics of legitimization when the entities involved challenge state power more explicitly—for instance, in the context of insurgent, terrorist, and paramilitary organizations.

Additionally, although we tested L-SP in diverse national contexts, the question of whether the scale can be employed in additional languages and geographical settings remains open. Our model indicated that partial scalar invariance was achieved by freeing the intercept of nine items, suggesting the need to revise these items to increase consistency in their interpretation across countries. Moreover, future research should aim to explore the scale in a broader range of cultural and sociopolitical environments and among diverse social groups. Multilevel models across a wider range of countries or regions might be employed to explore the relationship between structural factors and individuals’ appraisals of secret power. Longitudinal designs could be used to address the directionality of the relationship between constructs and changes in the appraisals of secret power over time.

## Conclusions

The study of secret power offers new avenues for understanding the complex dynamics of authority and legitimacy across communities and societies. It challenges and expands current psychological theories to accommodate the phenomenon of power exerted outside legal institutions. The L-SP scale allows investigating such dynamics comparatively across settings, tapping into participants’ general orientations towards criminal governance. However, when cross-country comparisons are not the main focus of the research, the scale can be calibrated toward specific groups and actors by modifying the instructions or the items’ form. Moreover, the supplemental material includes additional analyses testing a shorter version of the scale designed for inclusion in surveys with limited space (see Section D). While the longer version of the scale offers a more detailed measurement of criminal groups’ secret power, the shorter version provides a practical alternative for inclusion in broader studies where length is an issue. Both versions of the scale constitute valuable tools for future research, offering new instruments to investigate secret power in varied research settings and contexts.

## Supplemental Material

sj-docx-1-gpi-10.1177_13684302241290935 – Supplemental material for The psychology of criminal authority: Introducing the Legitimacy of Secret Power Scale Supplemental material, sj-docx-1-gpi-10.1177_13684302241290935 for The psychology of criminal authority: Introducing the Legitimacy of Secret Power Scale by Giovanni A. Travaglino, Alberto Mirisola, Chanki Moon, Pascal Burgmer, Hirotaka Imada, Isabella Giammusso, Silvana D’Ottone, Kengo Nawata, Miki Ozeki and Dominic Abrams in Group Processes & Intergroup Relations

## Notes

1. In this and all subsequent analyses, we accounted for reversed items in the scale by allowing these items to covary. Notably, the fit of measurement models without covariances among reversed items remained adequate. Models without covariances are described in the Supplemental Material.

2. Analyses for the measurement stability of the other measures in the model are presented in the Supplemental Material (p. 31). There was evidence for metric stability in both measures, which is necessary to compare relationships across samples.
