# Supplementary material for: The psychology of criminal authority: Introducing the Legitimacy of Secret Power Scale
Source: Group Process Intergroup Relat. 2024 Dec 8;28(3):477–503. doi: 10.1177/13684302241290935 (PMC11928288; doi:10.1177/13684302241290935)
Supplement: sj-docx-1-gpi-10.1177_13684302241290935 – Supplemental material for The psychology of criminal authority: Introducing the Legitimacy of Secret Power Scale [file sj-docx-1-gpi-10.1177_13684302241290935.docx]

The Psychology of Criminal Authority: Introducing the Legitimacy of Secret Power Scale

Supplemental Materials

Giovanni A. Travaglino^1^, Alberto Mirisola^2^, Chanki Moon^1^, Pascal Burgmer^3^, Hirotaka Imada^1,4^, Isabella Giammusso^2^, Silvana D'Ottone^5^, Kengo Nawata^6^, Miki Ozeki^7^, and Dominic Abrams^8^

^1^ Institute for the Study of Power, Crime, and Society, Department of Law and Criminology, Royal Holloway University of London, UK

^2^ Department of Psychology, Educational Science and Human Movement, University of Palermo, 90128, Palermo, Italy

^3^ School of Psychology, University of Southampton, Southampton, UK

^4^ Department of Psychology, Royal Holloway University of London, UK

^5^ School of Psychology, Pontificia Universidad Católica de Chile, Chile

^6^ Faculty of Humanities, Fukuoka University, Japan

^7^ Faculty of Humanities and Social Sciences, Okayama University, Japan

^8^ School of Psychology, University of Kent, UK

[**SECTION A: Study 1**](#_Section_A:_Study)

[**SECTION B: Study 2**](#_Section_B:_Study)

[**SECTION C: Study 3**](#_Section_C:_Study)

**[SECTION D: Short-Scale Development](#_Section_D:_Testing)**

# Section A: Study 1

Table A. Standardised factor loadings for the one-factor model with covariances among reversed items in Study 1

| **Indicator** | **Loadings** | ***p*** | **Lower.CI** | **Upper.CI** | **SE** | **z** |
| --- | --- | --- | --- | --- | --- | --- |
| L-SP19 | .777 | < .001 | .718 | .835 | .030 | 25.902 |
| L-SP3 | .768 | < .001 | .719 | .817 | .025 | 3.934 |
| L-SP17 | .747 | < .001 | .696 | .798 | .026 | 28.587 |
| L-SP27 | .738 | < .001 | .684 | .791 | .027 | 26.974 |
| L-SP5 | .734 | < .001 | .666 | .801 | .034 | 21.316 |
| L-SP1 | .730 | < .001 | .654 | .805 | .038 | 19.019 |
| L-SP2 | .723 | < .001 | .662 | .784 | .031 | 23.318 |
| L-SP32 | .720 | < .001 | .659 | .781 | .031 | 23.131 |
| L-SP28 | .703 | < .001 | .646 | .760 | .029 | 24.143 |
| L-SP4 | .699 | < .001 | .638 | .760 | .031 | 22.358 |
| L-SP40 | .694 | < .001 | .622 | .767 | .037 | 18.695 |
| L-SP30 | .682 | < .001 | .625 | .738 | .029 | 23.594 |
| L-SP38 | .670 | < .001 | .604 | .737 | .034 | 19.791 |
| L-SP24 | .670 | < .001 | .605 | .734 | .033 | 2.400 |
| L-SP7 | .657 | < .001 | .593 | .722 | .033 | 19.980 |
| L-SP22_R | .613 | < .001 | .538 | .688 | .038 | 15.933 |
| L-SP10_R | .550 | < .001 | .469 | .631 | .041 | 13.288 |
| L-SP21_R | .544 | < .001 | .447 | .640 | .049 | 11.072 |
| L-SP34_R | .455 | < .001 | .346 | .564 | .056 | 8.183 |
| L-SP13_R | .454 | < .001 | .362 | .545 | .047 | 9.742 |

The 20-item scale demonstrated adequate fit when the covariances of the five reverse items were constrained to 0, CFI = .93, RMSEA = .07, and SRMR = .051. Standardized Factor Loadings for this model are summarised in Table B below.

Table B. Standardised factor loadings for the one-factor model without covariances among reversed items in Study 1

| **Indicator** | **Loadings** | ***p*** | **Lower.CI** | **Upper.CI** | **SE** | **z** |
| --- | --- | --- | --- | --- | --- | --- |
| L-SP19 | .778 | < .001 | .721 | .835 | .029 | 26.741 |
| L-SP3 | .760 | < .001 | .711 | .810 | .025 | 3.245 |
| L-SP17 | .748 | < .001 | .698 | .799 | .026 | 29.114 |
| L-SP5 | .739 | < .001 | .673 | .805 | .034 | 21.862 |
| L-SP27 | .737 | < .001 | .683 | .790 | .027 | 26.965 |
| L-SP1 | .727 | < .001 | .654 | .800 | .037 | 19.450 |
| L-SP2 | .716 | < .001 | .653 | .778 | .032 | 22.563 |
| L-SP32 | .714 | < .001 | .653 | .775 | .031 | 22.951 |
| L-SP28 | .700 | < .001 | .643 | .758 | .029 | 23.969 |
| L-SP40 | .698 | < .001 | .625 | .770 | .037 | 18.882 |
| L-SP4 | .694 | < .001 | .632 | .755 | .031 | 22.043 |
| L-SP30 | .675 | < .001 | .617 | .732 | .029 | 22.929 |
| L-SP38 | .671 | < .001 | .604 | .737 | .034 | 19.859 |
| L-SP24 | .670 | < .001 | .606 | .733 | .033 | 2.596 |
| L-SP7 | .646 | < .001 | .579 | .713 | .034 | 18.954 |
| L-SP22_R | .636 | < .001 | .561 | .711 | .038 | 16.664 |
| L-SP21_R | .574 | < .001 | .479 | .669 | .049 | 11.826 |
| L-SP10_R | .570 | < .001 | .490 | .649 | .041 | 14.006 |
| L-SP13_R | .488 | < .001 | .399 | .578 | .046 | 1.682 |
| L-SP34_R | .482 | < .001 | .372 | .591 | .056 | 8.622 |

Table C. Exact p-values (in parentheses) for correlations in Table 2 (Study 1)

|  | 1 | 2 | 3 | 4 | 5 |
| --- | --- | --- | --- | --- | --- |
| (1) L-SP |  |  |  |  |  |
| (2) Masculine Honor | 0.255  (< .001) |  |  |  |  |
| (3) Willingness to Report Criminal Groups Activities | -0.353  (< .001) | -0.156  (.002) |  |  |  |
| (4) Age | -0.213  (< .001) | -0.129  (.011) | 0.191  (< .001) |  |  |
| (5) Gender | 0.070  (.163) | -0.251  (< .001) | -0.040  (.423) | 0.001  (.987) |  |

Note. *N* was 397 for all correlations except those involving age, which had an N of 387.

# Section B: Study 2

Table D. Demographic characteristics for the sample (*N* = 501) in Study 2

| **Demographic characteristic** | **Frequencies** | **Percentage**  **%** | **Cumulative Percentage %** |
| --- | --- | --- | --- |
| **Gender** |  |  |  |
| Male | 248 | 49.5 | - |
| Female | 247 | 49.3 | 98.8 |
| Non-Binary/Third Gender | 5 | 1 | 99.8 |
| Prefer not to say | 1 | .2 | 100 |
|  |  |  |  |
| **Ethnicity** |  |  |  |
| White | 456 | 91.02 | - |
| White/Asian | 1 | .2 | 91.22 |
| Black/African/Caribbean | 7 | 1.4 | 92.62 |
| Asian (Indian, Pakistani, Bangladeshi, Chinese, any other Asian background) | 20 | 3.99 | 96.61 |
| Mixed: two or more ethnic groups | 11 | 2.2 | 98.81 |
| Other (Arab or any others) | 1 | .2 | 99.01 |
| Prefer not to say | 4 | .8 | 99.81 |
| Unknown/ Not answered | 1 | .2 | 10.01 |
|  |  |  |  |
| **Education** |  |  |  |
| Completed Primary School | 1 | .2 | - |
| Some Secondary | 7 | 1.4 | 1.6 |
| Completed Secondary School | 98 | 19.56 | 21.16 |
| Vocational or Similar | 90 | 17.96 | 39.12 |
| Some University but no degree | 33 | 6.59 | 45.71 |
| University Bachelors Degree | 192 | 38.32 | 84.03 |
| Graduate or professional degree (MA, MS, MBA, PhD, JD, MD, DDS) | 75 | 14.97 | 99 |
| Unknown/ Not answered | 5 | 1 | 100 |
|  |  |  |  |
| **Student Status** |  |  |  |
| Student | 34 | 6.79 | - |
| Non-Student | 467 | 93.21 | 100 |
|  |  |  |  |
| **Region of residence** |  |  |  |
| North East England | 21 | 4.19 | - |
| North West England | 59 | 11.78 | 15.97 |
| Yorkshire and the Humber, England | 60 | 11.98 | 27.95 |
| East Midlands, England | 32 | 6.39 | 34.34 |
| West Midlands, England | 43 | 8.58 | 42.92 |
| East of England | 40 | 7.98 | 5.9 |
| London, England | 50 | 9.98 | 6.88 |
| South East, England | 74 | 14.77 | 75.65 |
| South West, England | 45 | 8.98 | 84.63 |
| Wales | 18 | 3.59 | 88.22 |
| Scotland | 38 | 7.58 | 95.8 |
| Northern Ireland | 7 | 1.4 | 97.3 |
| Unknown/ Not answered | 14 | 2.79 | 100 |
|  |  |  |  |
| **Employment** |  |  |  |
| Working (paid employee) | 314 | 62.67 | - |
| Working (self-employed) | 46 | 9.18 | 71.85 |
| Not working (temporary layoff from a job) | 2 | .4 | 72.25 |
| Not working (looking for work) | 31 | 6.19 | 78.44 |
| Not working (retired) | 57 | 11.38 | 89.82 |
| Not working (disabled) | 15 | 2.99 | 92.81 |
| Not working (other) | 27 | 5.39 | 98.2 |
| Prefer not to answer | 9 | 1.8 | 100 |

Note. MA: Master of Arts, MS: Master of Science, MBA: Master of Business Administration, PhD: Doctor of Philosophy, JD: Juris Doctor, MD: Doctor of Medicine, DDS: Doctor of Dental Surgery.

Table E. Standardised factor loadings for the one-factor model with covariances among reversed items in Study 2

| **Indicator** | **Loadings** | **p** | **Lower.CI** | **Upper.CI** | **SE** | **z** |
| --- | --- | --- | --- | --- | --- | --- |
| LSP3 | .829 | < .001 | .794 | .863 | .018 | 47.250 |
| LSP32 | .794 | <.001 | .748 | .840 | .023 | 33.810 |
| LSP28 | .790 | < .001 | .750 | .831 | .021 | 38.249 |
| LSP27 | .785 | < .001 | .742 | .828 | .022 | 35.731 |
| LSP2 | .782 | < .001 | .738 | .827 | .023 | 34.124 |
| LSP1 | .768 | < .001 | .714 | .822 | .028 | 27.772 |
| LSP5 | .761 | < .001 | .705 | .816 | .028 | 26.913 |
| LSP19 | .752 | < .001 | .691 | .813 | .031 | 24.211 |
| LSP38 | .736 | < .001 | .682 | .790 | .028 | 26.677 |
| LSP17 | .733 | < .001 | .671 | .795 | .031 | 23.295 |
| LSP4 | .732 | < .001 | .679 | .785 | .027 | 26.950 |
| LSP7 | .711 | < .001 | .659 | .764 | .027 | 26.572 |
| LSP30 | .684 | < .001 | .628 | .739 | .028 | 24.093 |
| LSP24 | .674 | < .001 | .609 | .738 | .033 | 2.485 |
| LSP40 | .651 | < .001 | .582 | .721 | .036 | 18.289 |
| LSP10_R | .615 | < .001 | .527 | .703 | .045 | 13.755 |
| LSP22_R | .552 | < .001 | .459 | .645 | .048 | 11.601 |
| LSP21_R | .463 | < .001 | .353 | .572 | .056 | 8.266 |
| LSP13_R | .443 | < .001 | .320 | .567 | .063 | 7.024 |
| LSP34_R | .417 | < .001 | .292 | .541 | .063 | 6.565 |

In Study 2, the 20-item scale demonstrated adequate fit when the covariances of the five reverse items were constrained to 0, CFI = .91, RMSEA = .078, SRMR = .054. Standardised Factor Loadings for this model are summarised in Table F below.

Table F. Standardised factor loadings for the one-factor model without covariances among reversed items in Study 2

| **Indicator** | **Loadings** | ***p*** | **Lower.CI** | **Upper.CI** | **SE** | **z** |
| --- | --- | --- | --- | --- | --- | --- |
| LSP3 | .825 | < .001 | .790 | .859 | .017 | 47.156 |
| LSP32 | .790 | < .001 | .744 | .837 | .024 | 33.267 |
| LSP27 | .784 | < .001 | .742 | .827 | .022 | 35.988 |
| LSP28 | .784 | < .001 | .743 | .826 | .021 | 37.286 |
| LSP2 | .778 | < .001 | .732 | .823 | .023 | 33.376 |
| LSP1 | .766 | < .001 | .712 | .821 | .028 | 27.491 |
| LSP5 | .764 | < .001 | .708 | .819 | .028 | 27.131 |
| LSP19 | .753 | < .001 | .692 | .814 | .031 | 24.243 |
| LSP17 | .736 | < .001 | .676 | .796 | .031 | 23.945 |
| LSP38 | .736 | < .001 | .683 | .790 | .027 | 26.909 |
| LSP4 | .729 | < .001 | .675 | .783 | .027 | 26.514 |
| LSP7 | .703 | < .001 | .650 | .756 | .027 | 25.958 |
| LSP30 | .679 | < .001 | .623 | .735 | .028 | 23.866 |
| LSP24 | .675 | < .001 | .610 | .739 | .033 | 2.528 |
| LSP40 | .655 | < .001 | .586 | .725 | .035 | 18.480 |
| LSP10_R | .634 | < .001 | .550 | .718 | .043 | 14.827 |
| LSP22_R | .573 | < .001 | .483 | .663 | .046 | 12.510 |
| LSP21_R | .494 | < .001 | .390 | .599 | .053 | 9.257 |
| LSP13_R | .473 | < .001 | .354 | .593 | .061 | 7.775 |
| LSP34_R | .442 | < .001 | .320 | .564 | .062 | 7.126 |

Table G. Correlations Among Variables in Study 2

|  | *1* | *2* | *3* | *4* | *5* | *6* | *7* | *8* | *9* | *10* | *11* | *12* | *13* | *14* | *15* | *16* |
| --- | --- | --- | --- | --- | --- | --- | --- | --- | --- | --- | --- | --- | --- | --- | --- | --- |
| L-SP (1) |  |  |  |  |  |  |  |  |  |  |  |  |  |  |  |  |
| Support for Extrajudicial Violence (2) | .317 *(<.001)* |  |  |  |  |  |  |  |  |  |  |  |  |  |  |  |
| Honor Ideology for Masculinity (3) | .332 *(<.001)* | .578 *(<.001)* |  |  |  |  |  |  |  |  |  |  |  |  |  |  |
| Attitudes towards Democracy (4) | -.272 *(<.001)* | -.226 *(<.001)* | -.196 *(<.001)* |  |  |  |  |  |  |  |  |  |  |  |  |  |
| Willingness to Report Criminal Group Activity (5) | -.333 *(<.001)* | -.165 *(<.001)* | -.141 *(.002)* | .163 *(<.001)* |  |  |  |  |  |  |  |  |  |  |  |  |
| General Aggressive Tendencies (6) | .161 *(<.001)* | .243 *(<.001)* | .218 *(<.001)* | -.136 *(.003)* | -.093 *(.041)* |  |  |  |  |  |  |  |  |  |  |  |
| Legal Cynicism (7) | .412 *(<.001)* | .304 *(<.001)* | .372 *(<.001)* | -.286 *(<.001)* | -.217 *(<.001)* | .178 *(<.001)* |  |  |  |  |  |  |  |  |  |  |
| Legitimacy of the Police (8) | -.217 *(<.001)* | -.212 *(<.001)* | -.089 *(.049)* | .209 *(<.001)* | .319 *(<.001)* | -.115 *(.011)* | -.278 *(<.001)* |  |  |  |  |  |  |  |  |  |
| Social Threat (9) | -.383 *(<.001)* | -.041 *(.362)* | .017 *(.712)* | .037 *(.410)* | .218 *(<.001)* | -.030 *(.515)* | -.141 *(.002)* | .045 *(.319)* |  |  |  |  |  |  |  |  |
| Fear of Criminal Groups (10) | -.167 *(<.001)* | -.106 *(.019)* | -.118 *(.009)* | .011 *(.815)* | .137 *(.002)* | .097 *(.033)* | -.077 *(.089)* | .130 *(.004)* | .340 *(<.001)* |  |  |  |  |  |  |  |
| Social Desirability (11) | -.131 *(.004)* | -.129 *(.004)* | -.059 *(.197)* | -.029 *(.517)* | .111 *(.014)* | -.299 *(<.001)* | -.119 *(.009)* | .093 *(.041)* | .113 *(.012)* | -.045 *(.321)* |  |  |  |  |  |  |
| Gender (12) | -.018 *(.694)* | -.258 *(<.001)* | -.350 *(<.001)* | -.045 *(.321)* | .019 *(.677)* | .035 *(.449)* | -.078 *(.089)* | .045 *(.323)* | .068 *(.135)* | .218 *(<.001)* | .088 *(.053)* |  |  |  |  |  |
| Age (13) | -.258 *(<.001)* | -.227 *(<.001)* | -.067 *(.138)* | .139 *(.002)* | .194 *(<.001)* | -.177 *(<.001)* | -.147 *(.001)* | .252 *(<.001)* | .245 *(<.001)* | .010 *(.826)* | .205 *(<.001)* | -.04 *(.390)* |  |  |  |  |
|  |  |  |  |  |  |  |  |  |  |  |  |  |  |  |  |  |
|  | *1* | *2* | *3* | *4* | *5* | *6* | *7* | *8* | *9* | *10* | *11* | *12* | *13* | *14* | *15* | *16* |
| Education (14) | .002 *(.962)* | -.017 *(.706)* | -.109 *(.017)* | .182 *(<.001)* | .111 *(.015)* | -.060 *(.187)* | -.035 *(.439)* | -.041 *(.372)* | -.127 *(.005)* | -.093 *(.041)* | -.151 *(.001)* | -.01 *(.949)* | -.119 *(.009)* |  |  |  |
| Employment (15) | -.058 *(.205)* | -.097 *(.034)* | -.049 *(.286)* | -.006 *(.897)* | .060 *(.190)* | -.006 *(.903)* | -.039 *(.397)* | .090 *(.049)* | .022 *(.631)* | .027 *(.557)* | .092 *(.044)* | .078 *(.090)* | .297 *(<.001)* | -.151 *(.001)* |  |  |
| Subjective Social Status (16) | .099 *(.028)* | .109 *(.016)* | .082 *(.069)* | -.164 *(<.001)* | -.232 *(<.001)* | .168 *(<.001)* | .181 *(<.001)* | -.219 *(<.001)* | -.030 *(.515)* | -.027 *(.548)* | -.069 *(.130)* | -.039 *(.395)* | -.139 *(.002)* | -.313 *(<.001)* | .115 *(.012)* |  |
| Political Orientation | -.022 *(.622)* | .143 *(.002)* | .301 *(<.001)* | -.154 *(.001)* | .058 *(.198)* | -.032 *(.481)* | -.006 *(.894)* | .166 *(<.001)* | .134 *(.003)* | .027 *(.556)* | .118 *(.009)* | -.132 *(.004)* | .244 *(<.001)* | -.164 *(<.001)* | .085 *(.064)* | -.088 *(.052)* |

Note. L-PS: Legitimacy of Secret Power Scale. P-values appear in parentheses. Gender: 1 = male, 2 = female, Employment: 1 = Employed, 2 = Unemployed.

# Section C: Study 3

Table H. Demographic characteristics for the UK sample (*N* = 582) in Study 3

| **Demographic characteristic** | **Frequencies** | **Percentage**  **%** | **Cumulative Percentage %** |
| --- | --- | --- | --- |
| **Gender** |  |  |  |
| Male | 272 | 46.74 | - |
| Female | 303 | 52.06 | 98.80 |
| Non-Binary/Third Gender | 2 | .34 | 99.14 |
| Prefer not to say | 5 | .86 | 100 |
|  |  |  |  |
| **Ethnicity** |  |  |  |
| White | 505 | 86.77 | - |
| Black/African/Caribbean | 26 | 4.47 | 91.24 |
| Asian (Indian, Pakistani, Bangladeshi, Chinese, any other Asian background) | 26 | 4.47 | 95.71 |
| Mixed: two or more ethnic groups | 21 | 3.61 | 99.32 |
| Other (Arab or any others) | 2 | .34 | 99.66 |
| Prefer not to say | 2 | .34 | 100 |
|  |  |  |  |
| **Education** |  |  |  |
| Some Primary School | 5 | .86 | - |
| Completed Primary School | 2 | .34 | 1.20 |
| Some Secondary | 19 | 3.26 | 4.46 |
| Completed Secondary School | 204 | 35.05 | 39.51 |
| Vocational or Similar | 116 | 19.93 | 59.44 |
| Some University but no degree | 45 | 7.73 | 67.17 |
| University Bachelors Degree | 133 | 22.85 | 90.02 |
| Graduate or professional degree (MA, MS, MBA, PhD, JD, MD, DDS) | 50 | 8.59 | 98.61 |
| Prefer not to say | 8 | 1.37 | 99.98 |
|  |  |  |  |
| **Student Status** |  |  |  |
| Student | 56 | 9.62 | - |
| Non-Student | 526 | 90.38 | 100 |
|  |  |  |  |
| **Region of residence** |  |  |  |
| North East England | 37 | 6.36 | - |
| North West England | 91 | 15.64 | 22.00 |
| Yorkshire and the Humber, England | 51 | 8.76 | 30.76 |
| East Midlands, England | 48 | 8.25 | 39.01 |
| West Midlands, England | 55 | 9.45 | 48.46 |
| East of England | 47 | 8.08 | 56.54 |
| London, England | 62 | 10.65 | 67.19 |
| South East, England | 74 | 12.71 | 79.90 |
| South West, England | 36 | 6.19 | 86.09 |
| Wales | 30 | 5.15 | 91.24 |
| Scotland | 35 | 6.01 | 97.25 |
| Northern Ireland | 16 | 2.75 | 100 |
|  |  |  |  |
| **Employment** |  |  |  |
| Working (paid employee) | 376 | 64.60 | - |
| Working (self-employed) | 41 | 7.04 | 71.64 |
| Not working (temporary layoff from a job) | 7 | 1.20 | 72.84 |
| Not working (looking for work) | 39 | 3.70 | 79.54 |
| Not working (retired) | 31 | 5.33 | 84.87 |
| Not working (disabled) | 52 | 8.93 | 93.80 |
| Not working (other) | 36 | 6.19 | 99.99 |
|  |  |  |  |
| **Income** |  |  |  |
| Less than £20k | 131 | 22.51 | - |
| £20-39,999 | 202 | 34.71 | 57.22 |
| £40-59,999 | 112 | 19.24 | 76.46 |
| £60-99,999 | 82 | 14.09 | 90.55 |
| More than £100K | 16 | 2.75 | 93.30 |
| Prefer not to say | 39 | 6.70 | 100 |

Note. MA: Master of Arts, MS: Master of Science, MBA: Master of Business Administration, PhD: Doctor of Philosophy, JD: Juris Doctor, MD: Doctor of Medicine, DDS: Doctor of Dental Surgery.

Table I. Demographic characteristics for the US sample (*N* = 604) in Study 3

| **Demographic characteristic** | **Frequencies** | **Percentage**  **%** | **Cumulative Percentage %** |
| --- | --- | --- | --- |
| **Gender** |  |  |  |
| Male | 275 | 45.53 | - |
| Female | 328 | 54.30 | 99.83 |
| Non-Binary/Third Gender | 1 | .17 | 100 |
|  |  |  |  |
| **Ethnicity** |  |  |  |
| White or Caucasian | 467 | 77.32 | - |
| Black/African/Caribbean | 79 | 13.08 | 90.40 |
| Asian (Indian, Pakistani, Bangladeshi, Chinese, any other Asian background) | 15 | 2.48 | 92.88 |
| Mixed: two or more ethnic groups | 18 | 2.98 | 95.86 |
| Native Hawaiian or Other Pacific Islander | 1 | 0.17 | 96.03 |
| American Indian/Native American or Alaska Native | 5 | 0.83 | 96.86 |
| Other (Arab or any others) | 19 | 3.15 | 100.01 |
|  |  |  |  |
| **Education** |  |  |  |
| Some high school or less | 21 | 3.48 | - |
| High school diploma or GED | 136 | 22.52 | 26.00 |
| Some college, but no degree | 123 | 20.36 | 46.36 |
| Associates or technical degree | 63 | 10.43 | 56.79 |
| University Bachelors Degree | 137 | 22.68 | 79.47 |
| Graduate or professional degree (MA, MS, MBA, PhD, JD, MD, DDS) | 124 | 20.53 | 100 |
|  |  |  |  |
| **Student Status** |  |  |  |
| Student | 53 | 8.77 | - |
| Non-Student | 551 | 91.23 | 100 |
|  |  |  |  |
| **Region of residence** |  |  |  |
| Midwest | 111 | 18.38 | - |
| Northeast | 145 | 24.01 | 42.39 |
| South | 237 | 39.24 | 81.63 |
| West | 111 | 18.38 | 100.01 |
|  |  |  |  |
| **Employment** |  |  |  |
| Working (paid employee) | 309 | 51.16 | - |
| Working (self-employed) | 60 | 9.93 | 61.09 |
| Not working (temporary layoff from a job) | 5 | 0.83 | 61.92 |
| Not working (looking for work) | 53 | 8.77 | 70.69 |
| Not working (retired) | 81 | 13.41 | 84.10 |
| Not working (disabled) | 48 | 7.95 | 92.05 |
| Not working (other) | 48 | 7.95 | 100 |
|  |  |  |  |
| **Income** |  |  |  |
| Less than $25,000 | 156 | 25.83 | - |
| $25,000-$49,999 | 120 | 19.87 | 45.70 |
| $50,000-$74,999 | 86 | 14.24 | 59.94 |
| $75,000-$99,999 | 57 | 9.44 | 69.38 |
| $100,000-$149,999 | 82 | 13.58 | 82.96 |
| $150,000 or more | 91 | 15.07 | 98.03 |
| Prefer not to say | 12 | 1.99 | 100.02 |

Note. GED: General Educational Development, MA: Master of Arts, MS: Master of Science, MBA: Master of Business Administration, PhD: Doctor of Philosophy, JD: Juris Doctor, MD: Doctor of Medicine, DDS: Doctor of Dental Surgery.

Table J. Demographic characteristics for Italian sample (*N* = 600) in Study 3

| **Demographic characteristic** | **Frequencies** | **Percentage**  **%** | **Cumulative Percentage %** |
| --- | --- | --- | --- |
| **Gender** |  |  |  |
| Male | 296 | 49.33 | - |
| Female | 301 | 50.17 | 99.50 |
| Non-Binary/Third Gender | 1 | 0.17 | 99.67 |
| Prefer not to say | 2 | 0.33 | 100 |
|  |  |  |  |
| **Ethnicity** |  |  |  |
| Italian | 578 | 96.33 | - |
| Mixed two or more ethnic groups | 6 | 1.00 | 97.33 |
| Albanian | 3 | .50 | 97.83 |
| Romanian | 5 | .83 | 98.66 |
| Other European | 3 | .50 | 99.16 |
| African | 3 | .50 | 99.66 |
| Other minority | 2 | .33 | 99.99 |
|  |  |  |  |
| **Education** |  |  |  |
| Some Primary School | 2 | .33 | - |
| Completed Primary School | 23 | 3.83 | 4.16 |
| Some Secondary | 26 | 4.33 | 8.49 |
| Completed Secondary School | 230 | 38.33 | 46.82 |
| Vocational or Similar | 62 | 10.33 | 57.15 |
| Some University but no degree | 60 | 10.00 | 67.15 |
| University Bachelors Degree | 74 | 12.33 | 79.48 |
| Graduate or professional degree (MA, MS, MBA, PhD, JD, MD, DDS) | 119 | 19.83 | 99.31 |
| Prefer not to say | 4 | 0.67 | 99.98 |
|  |  |  |  |
| **Student Status** |  |  |  |
| Student | 60 | 10.00 | - |
| Non-Student | 540 | 90.00 | 100 |
|  |  |  |  |
| **Region of residence** |  |  |  |
| Center | 11 | 18.50 | - |
| Islands | 74 | 12.33 | 30.83 |
| North East | 112 | 18.67 | 49.50 |
| North West | 168 | 28.00 | 77.50 |
| South | 135 | 22.50 | 100 |
|  |  |  |  |
| **Employment** |  |  |  |
| Working (paid employee) | 325 | 54.17 | - |
| Working (self-employed) | 94 | 15.67 | 69.84 |
| Not working (temporary layoff from a job) | 10 | 1.67 | 71.51 |
| Not working (looking for work) | 49 | 8.17 | 79.68 |
| Not working (retired) | 49 | 8.17 | 87.85 |
| Not working (disabled) | 4 | .67 | 88.52 |
| Not working (other) | 69 | 11.50 | 100.02 |
|  |  |  |  |
| **Income** |  |  |  |
| Less than 25,000 Euros | 211 | 35.17 | - |
| 25,000 - 49,999 Euros | 251 | 41.83 | 77.00 |
| 50,000 - 99,999 Euros | 69 | 11.50 | 88.50 |
| 100,000 - 199,999 Euros | 7 | 1.17 | 89.67 |
| More than 200,000 Euros | 3 | .50 | 90.17 |
| Prefer not to say | 59 | 9.83 | 100 |

Note. MA: Master of Arts, MS: Master of Science, MBA: Master of Business Administration, PhD: Doctor of Philosophy, JD: Juris Doctor, MD: Doctor of Medicine, DDS: Doctor of Dental Surgery.

Table K. Demographic characteristics for Japanese sample (*N* = 603) in Study 3

| **Demographic characteristic** | **Frequencies** | **Percentage**  **%** | **Cumulative Percentage %** |
| --- | --- | --- | --- |
| **Gender** |  |  |  |
| Male | 302 | 50.08 | - |
| Female | 298 | 49.42 | 99.50 |
| Non-Binary/Third Gender | 2 | .33 | 99.83 |
| Prefer not to say | 1 | .17 | 100 |
|  |  |  |  |
| **Residency status** |  |  |  |
| Japanese Citizen | 593 | 98.34 | - |
| Japanese Permanent Resident | 7 | 1.16 | 99.50 |
| Foreign/Expatriate | 1 | .17 | 99.67 |
| Other | 2 | .33 | 100 |
|  |  |  |  |
| **Education** |  |  |  |
| Secondary Completed | 15 | 2.49 | - |
| Some high school | 63 | 10.45 | 12.94 |
| High School Completed | 124 | 20.56 | 33.50 |
| Vocational or Similar | 113 | 18.74 | 52.24 |
| Some University but no degree | 9 | 1.49 | 53.73 |
| University Bachelors Degree | 245 | 40.63 | 94.36 |
| Graduate or professional degree (MA, MS, MBA, PhD, JD, MD, DDS) | 32 | 5.31 | 99.67 |
| Prefer not to say | 2 | .33 | 100 |
|  |  |  |  |
| **Student Status** |  |  |  |
| Student | 57 | 9.45 | - |
| Non-Student | 546 | 90.55 | 100 |
|  |  |  |  |
| **Region of residence** |  |  |  |
| Chubu | 83 | 13.76 | - |
| Chugoku | 30 | 4.98 | 18.74 |
| Hokkaido | 38 | 6.30 | 25.04 |
| Kansai | 115 | 19.07 | 44.11 |
| Kanto | 247 | 40.96 | 85.07 |
| Kyushu | 45 | 7.46 | 92.53 |
| Shikoku | 17 | 2.82 | 95.35 |
| Tohoku | 28 | 4.64 | 99.99 |
|  |  |  |  |
| **Employment** |  |  |  |
| Working (paid employee) | 391 | 64.84 | - |
| Working (self-employed) | 53 | 8.79 | 73.63 |
| Not working (temporary layoff from a job) | 6 | 1.00 | 74.63 |
| Not working (looking for work) | 34 | 5.64 | 80.27 |
| Not working (retired) | 47 | 7.79 | 88.06 |
| Not working (disabled) | 24 | 3.98 | 92.04 |
| Not working (other) | 48 | 7.96 | 100 |
|  |  |  |  |
| **Income** |  |  |  |
| ¥0 - ¥199 (x ¥10,000) | 82 | 13.60 | - |
| ¥200 - ¥399 (x ¥10,000) | 127 | 21.06 | 34.66 |
| ¥400 - ¥599 (x ¥10,000) | 116 | 19.24 | 53.90 |
| ¥600 - ¥799 (x ¥10,000) | 77 | 12.77 | 66.67 |
| ¥800 - ¥999 (x ¥10,000) | 55 | 9.12 | 75.79 |
| ¥1000+ (x ¥10,000) or more | 59 | 9.78 | 85.57 |
| Prefer not to say | 87 | 14.43 | 100 |

Note. MA: Master of Arts, MS: Master of Science, MBA: Master of Business Administration, PhD: Doctor of Philosophy, JD: Juris Doctor, MD: Doctor of Medicine, DDS: Doctor of Dental Surgery.

In Study 3, the 20-item scale demonstrated adequate fit when the covariances of the five reverse items were constrained to 0, as shown in Table L below.

Table L. Measurement model fit indices across countries in Study 3 without covariances

| **Country** | **CFI** | **RMSEA** | **SRMR** |
| --- | --- | --- | --- |
| UK | 0.934 | 0.067 | 0.051 |
| US | 0.943 | 0.066 | 0.047 |
| Italy | 0.947 | 0.057 | 0.044 |
| Japan | 0.958 | 0.050 | 0.042 |

The standardized loadings across countries are reported in Table M below.

Table M. Standardized Factor Loadings across countries in Study 3 without covariances among reversed items

| **Items** | **λ** | | | |
| --- | --- | --- | --- | --- |
|  | **UK** | **US** | **Italy** | **Japan** |
| L-SP1 | 0.81 | 0.81 | 0.69 | 0.71 |
| L-SP3 | 0.80 | 0.79 | 0.77 | 0.79 |
| L-SP27 | 0.79 | 0.78 | 0.86 | 0.74 |
| L-SP2 | 0.79 | 0.84 | 0.72 | 0.73 |
| L-SP19 | 0.79 | 0.78 | 0.75 | 0.78 |
| L-SP28 | 0.77 | 0.81 | 0.79 | 0.79 |
| L-SP4 | 0.77 | 0.83 | 0.67 | 0.69 |
| L-SP5 | 0.76 | 0.80 | 0.83 | 0.73 |
| L-SP32 | 0.75 | 0.79 | 0.78 | 0.79 |
| L-SP17 | 0.75 | 0.79 | 0.75 | 0.81 |
| L-SP7 | 0.74 | 0.72 | 0.74 | 0.76 |
| L-SP38 | 0.70 | 0.71 | 0.71 | 0.72 |
| L-SP40 | 0.69 | 0.79 | 0.73 | 0.66 |
| L-SP30 | 0.68 | 0.75 | 0.59 | 0.77 |
| L-SP24 | 0.64 | 0.67 | 0.63 | 0.57 |
| L-SP10_R | 0.58 | 0.53 | 0.41 | 0.50 |
| L-SP22_R | 0.56 | 0.48 | 0.43 | 0.58 |
| L-SP21_R | 0.55 | 0.51 | 0.45 | 0.23 |
| L-SP13_R | 0.43 | 0.45 | 0.40 | 0.30 |
| L-SP34_R | 0.41 | 0.52 | 0.39 | 0.17 |

We tested the measurement stability of the model with covariances among reversed items constrained to 0. In this version, L-SP had adequate configural fit, CFI = .945, RMSEA = .061, SRMR = .044. Constraining the loadings to be the same across groups did not significantly deteriorate the model’s fit, ΔCFI = -.003, ΔMcDonald’s NCI = - .013 with only the ΔGamma hat = -.003 indicating a slight discrepancy, suggesting that the measure achieved full metric invariance. Constraining the intercepts to be equal across groups resulted in a model misfit higher than the thresholds across indices, ΔCFI = -.018, ΔMcDonald’s NCI = - .06, ΔGamma hat = -.01. We, therefore, tested a partially invariant model in which we attempted to identify which of the intercepts were causing the misfit and allowed them to vary freely across countries. By releasing the constraints on the intercepts of nine items (Items: 30, 34, 21, 4, 19, 22, 7, 17, and 13), we achieved partial scalar invariance according to two of the three indices ΔCFI = -.003, ΔMcDonald’s NCI = - .016, with only the ΔGamma hat = -.004 indicating a slight discrepancy.

| Table N. Correlations among all variables in Study 3 (Italy) | | | | | | | | | | | | | | | | | |
| --- | --- | --- | --- | --- | --- | --- | --- | --- | --- | --- | --- | --- | --- | --- | --- | --- | --- |
|  | 1 | 2 | 3 | 4 | 5 | 6 | 7 | 8 | 8 | 10 | 11 | 12 | 13 | 14 | 15 | 16 | 17 |
| L-SP (1) |  |  |  |  |  |  |  |  |  |  |  |  |  |  |  |  |  |
| Masculine Honor (2) | 0.353 (<.001) |  |  |  |  |  |  |  |  |  |  |  |  |  |  |  |  |
| Democracy (3) | -0.339 (<.001) | -0.258 (<.001) |  |  |  |  |  |  |  |  |  |  |  |  |  |  |  |
| Cooperation Legal Authorities (4) | -0.304 (<.001) | -0.108 (.010) | 0.210 (<.001) |  |  |  |  |  |  |  |  |  |  |  |  |  |  |
| Legal Cynicism (5) | 0.395 (<.001) | 0.376 (<.001) | -0.191 (<.001) | -0.178 (<.001) |  |  |  |  |  |  |  |  |  |  |  |  |  |
| Legitimacy Police (6) | -0.218 (<.001) | -0.043 (.307) | 0.336 (<.001) | 0.279 (<.001) | -0.108 (.009) |  |  |  |  |  |  |  |  |  |  |  |  |
| National Threat (7) | -0.485 (<.001) | -0.104 (.012) | 0.159 (<.001) | 0.345 (<.001) | -0.198 (<.001) | 0.169 (<.001) |  |  |  |  |  |  |  |  |  |  |  |
| Fear (8) | -0.099 (.017) | -0.022 (.596) | 0.152 (<.001) | 0.079 (.057) | 0.045 (.278) | 0.144 (<.001) | 0.272 (<.001) |  |  |  |  |  |  |  |  |  |  |
| Anger (9) | -0.365 (<.001) | -0.080 (.054) | 0.287 (<.001) | 0.326 (<.001) | -0.141 (.001) | 0.268 (<.001) | 0.507 (<.001) | 0.393 (<.001) |  |  |  |  |  |  |  |  |  |
| Civic Honesty (10) | -0.535 (<.001) | -0.273 (<.001) | 0.294 (<.001) | 0.296 (<.001) | -0.376 (<.001) | 0.321 (<.001) | 0.347 (<.001) | 0.111 (.007) | 0.358 (<.001) |  |  |  |  |  |  |  |  |
| SDO (11) | 0.410 (<.001) | 0.344 (<.001) | -0.308 (<.001) | -0.208 (<.001) | 0.261 (<.001) | -0.117 (.005) | -0.220 (<.001) | -0.031 (.455) | -0.152 (<.001) | -0.268 (<.001) |  |  |  |  |  |  |  |
| Gender (12) | -0.087 (.036) | -0.155 (<.001) | 0.068 (.102) | -0.035 (.402) | 0.020 (.633) | 0.007 (.873) | 0.107 (.010) | 0.314 (<.001) | 0.086 (.039) | 0.127 (.002) | -0.133 (.001) |  |  |  |  |  |  |
| Age (13) | -0.208 (<.001) | -0.027 (.514) | 0.214 (<.001) | 0.118 (.005) | -0.110 (.008) | 0.278 (<.001) | 0.201 (<.001) | 0.021 (.610) | 0.218 (<.001) | 0.380 (<.001) | -0.001 (.984) | 0.019 (.655) |  |  |  |  |  |
| Education (14) | -0.064 (.127) | -0.173 (<.001) | 0.085 (.041) | 0.070 (.094) | -0.158 (<.001) | -0.113 (.007) | -0.026 (.536) | -0.017 (.676) | -0.013 (.763) | -0.049 (.244) | -0.171 (<.001) | 0.047 (.266) | -0.197 (<.001) |  |  |  |  |
|  | 1 | 2 | 3 | 4 | 5 | 6 | 7 | 8 | 8 | 10 | 11 | 12 | 13 | 14 | 15 | 16 | 17 |
| Employment (15) | -0.032 (.440) | -0.018 (.665) | -0.017 (.674) | -0.014 (.744) | -0.017 (.689) | 0.013 (.763) | 0.023 (.576) | 0.066 (.111) | 0.022 (.590) | 0.078 (.059) | 0.039 (.344) | 0.193 (<.001) | 0.158 (<.001) | -0.244 (<.001) |  |  |  |
| SES (16) | -0.063 (.130) | -0.010 (.810) | 0.003 (.942) | -0.055 (.184) | 0.012 (.765) | -0.068 (.101) | 0.008 (.848) | 0.018 (.668) | 0.015 (.727) | 0.071 (.087) | -0.043 (.299) | 0.034 (.417) | 0.153 (<.001) | -0.202 (<.001) | 0.165 (<.001) |  |  |
| Political Orient. (17) | 0.145 (<.001) | 0.245 (<.001) | -0.188 (<.001) | 0.018 (.670) | 0.141 (.001) | 0.162 (<.001) | 0.016 (.707) | 0.000 (.996) | 0.027 (.517) | -0.013 (.753) | 0.305 (<.001) | -0.048 (.247) | 0.106 (.010) | -0.189 (<.001) | -0.033 (.421) | -0.003 (.950) |  |
| Income | -0.065 (.138) | -0.050 (.256) | 0.092 (.035) | 0.124 (.004) | -0.141 (.001) | 0.020 (.644) | -0.011 (.797) | -0.044 (.320) | 0.028 (.521) | 0.032 (.464) | -0.065 (.137) | -0.127 (.004) | 0.016 (.712) | 0.197 (<.001) | -0.192 (<.001) | -0.353 (<.001) | -.047 (.287) |

Note. L-PS: Legitimacy of Secret Power Scale. P-values appear in parentheses. Gender: 1 = male, 2 = female, Employment: 1 = Employed, 2 = Unemployed.

| Table O. Correlations among all variables in Study 3 (United States) | | | | | | | | | | | | | | | | | |
| --- | --- | --- | --- | --- | --- | --- | --- | --- | --- | --- | --- | --- | --- | --- | --- | --- | --- |
|  | 1 | 2 | 3 | 4 | 5 | 6 | 7 | 8 | 8 | 10 | 11 | 12 | 13 | 14 | 15 | 16 | 17 |
| L-SP (1) |  |  |  |  |  |  |  |  |  |  |  |  |  |  |  |  |  |
| Masculine Honor (2) | 0.239 (<.001) |  |  |  |  |  |  |  |  |  |  |  |  |  |  |  |  |
| Democracy (3) | -0.260 (<.001) | 0.023 (.576) |  |  |  |  |  |  |  |  |  |  |  |  |  |  |  |
| Cooperation Legal Authorities (4) | -0.255 (<.001) | 0.042 (.314) | 0.284 (<.001) |  |  |  |  |  |  |  |  |  |  |  |  |  |  |
| Legal Cynicism (5) | 0.413 (<.001) | 0.456 (<.001) | -0.049 (.234) | -0.062 (.130) |  |  |  |  |  |  |  |  |  |  |  |  |  |
| Legitimacy Police (6) | -0.183 (<.001) | 0.156 (<.001) | 0.442 (<.001) | 0.479 (<.001) | -0.013 (.746) |  |  |  |  |  |  |  |  |  |  |  |  |
| National Threat (7) | -0.412 (<.001) | 0.058 (.157) | 0.266 (<.001) | 0.402 (<.001) | -0.081 (.049) | 0.345 (<.001) |  |  |  |  |  |  |  |  |  |  |  |
| Fear (8) | -0.175 (<.001) | -0.005 (.908) | 0.111 (.007) | 0.240 (<.001) | 0.065 (.117) | 0.224 (<.001) | 0.440 (<.001) |  |  |  |  |  |  |  |  |  |  |
| Anger (9) | -0.336 (<.001) | 0.072 (.080) | 0.221 (<.001) | 0.325 (<.001) | -0.030 (.462) | 0.272 (<.001) | 0.547 (<.001) | 0.537 (<.001) |  |  |  |  |  |  |  |  |  |
| Civic Honesty (10) | -0.413 (<.001) | -0.351 (<.001) | 0.067 (.103) | 0.105 (.011) | -0.500 (<.001) | 0.061 (.140) | 0.113 (.006) | -0.107 (.009) | 0.022 (.597) |  |  |  |  |  |  |  |  |
| SDO (11) | 0.221 (<.001) | 0.248 (<.001) | -0.212 (<.001) | -0.022 (.591) | 0.123 (.003) | 0.081 (.049) | -0.028 (.499) | -0.012 (.767) | 0.035 (.402) | -0.213 (<.001) |  |  |  |  |  |  |  |
| Gender (12) | 0.043 (.303) | -0.201 (<.001) | -0.209 (<.001) | -0.066 (.109) | -0.088 (.033) | -0.127 (.002) | -0.043 (.299) | 0.043 (.296) | -0.072 (.079) | 0.082 (.046) | -0.050 (.223) |  |  |  |  |  |  |
| Age (13) | -0.350 (<.001) | -0.245 (<.001) | 0.157 (<.001) | 0.143 (<.001) | -0.370 (<.001) | 0.173 (<.001) | 0.245 (<.001) | -0.026 (.534) | 0.141 (.001) | 0.406 (<.001) | -0.040 (.336) | -0.033 (.418) |  |  |  |  |  |
| Education (14) | -0.059 (.150) | 0.073 (.076) | 0.343 (<.001) | 0.151 (<.001) | 0.125 (.002) | 0.274 (<.001) | 0.089 (.031) | 0.152 (<.001) | 0.144 (<.001) | -0.129 (.002) | -0.050 (.223) | -0.201 (<.001) | -0.058 (.158) |  |  |  |  |
|  | 1 | 2 | 3 | 4 | 5 | 6 | 7 | 8 | 8 | 10 | 11 | 12 | 13 | 14 | 15 | 16 | 17 |
| Employment (15) | -0.069 (.093) | -0.142 (.001) | -0.111 (.007) | -0.047 (.253) | -0.202 (<.001) | -0.092 (.025) | 0.023 (.583) | -0.065 (.116) | -0.047 (.255) | 0.210 (<.001) | 0.002 (.955) | 0.170 (<.001) | 0.309 (<.001) | -0.411 (<.001) |  |  |  |
| SES (16) | -0.075 (.069) | -0.259 (<.001) | -0.248 (<.001) | -0.242 (<.001) | -0.203 (<.001) | -0.319 (<.001) | -0.075 (.070) | -0.043 (.296) | -0.106 (.010) | 0.213 (<.001) | -0.168 (<.001) | 0.111 (.007) | 0.096 (.020) | -0.388 (<.001) | 0.272 (<.001) |  |  |
| Political Orient. (17) | 0.082 (.048) | 0.358 (<.001) | 0.022 (.600) | 0.172 (<.001) | 0.111 (.007) | 0.265 (<.001) | 0.107 (.010) | 0.055 (.184) | 0.114 (.006) | -0.089 (.030) | 0.377 (<.001) | -0.094 (.023) | -0.068 (.100) | 0.040 (.338) | -0.043 (.299) | -0.262 (<.001) |  |
| Income | -0.051 (.219) | 0.131 (.002) | 0.294 (<.001) | 0.218 (<.001) | 0.125 (.003) | 0.282 (<.001) | 0.103 (.013) | 0.163 (<.001) | 0.203 (<.001) | -0.099 (.017) | -0.013 (.758) | -0.152 (<.001) | -0.122 (.003) | 0.586 (<.001) | -0.450 (<.001) | -0.505 (<.001) | 0.104 (.012) |

Note. L-PS: Legitimacy of Secret Power Scale. P-values appear in parentheses. Gender: 1 = male, 2 = female, Employment: 1 = Employed, 2 = Unemployed.

| Table P. Correlations among all variables in Study 3 (United Kingdom) | | | | | | | | | | | | | | | | | |
| --- | --- | --- | --- | --- | --- | --- | --- | --- | --- | --- | --- | --- | --- | --- | --- | --- | --- |
|  | 1 | 2 | 3 | 4 | 5 | 6 | 7 | 8 | 9 | 10 | 11 | 12 | 13 | 14 | 15 | 16 | 17 |
| L-SP (1) |  |  |  |  |  |  |  |  |  |  |  |  |  |  |  |  |  |
| Masculine Honor (2) | 0.296 (<.001) |  |  |  |  |  |  |  |  |  |  |  |  |  |  |  |  |
| Democracy (3) | -0.326 (<.001) | -0.188 (<.001) |  |  |  |  |  |  |  |  |  |  |  |  |  |  |  |
| Cooperation Legal Authorities (4) | -0.363 (<.001) | -0.132 (.002) | 0.185 (<.001) |  |  |  |  |  |  |  |  |  |  |  |  |  |  |
| Legal Cynicism (5) | 0.417 (<.001) | 0.387 (<.001) | -0.268 (<.001) | -0.281 (<.001) |  |  |  |  |  |  |  |  |  |  |  |  |  |
| Legitimacy Police (6) | -0.160 (<.001) | -0.044 (.309) | 0.331 (<.001) | 0.409 (<.001) | -0.236 (<.001) |  |  |  |  |  |  |  |  |  |  |  |  |
| National Threat (7) | -0.414 (<.001) | -0.036 (.400) | 0.179 (<.001) | 0.282 (<.001) | -0.179 (<.001) | 0.166 (<.001) |  |  |  |  |  |  |  |  |  |  |  |
| Fear (8) | -0.099 (.021) | -0.098 (.021) | 0.022 (.602) | 0.187 (<.001) | -0.096 (.026) | 0.195 (<.001) | 0.371 (<.001) |  |  |  |  |  |  |  |  |  |  |
| Anger (9) | -0.362 (<.001) | -0.108 (.012) | 0.205 (<.001) | 0.342 (<.001) | -0.256 (<.001) | 0.259 (<.001) | 0.470 (<.001) | 0.496 (<.001) |  |  |  |  |  |  |  |  |  |
| Civic Honesty (10) | -0.386 (<.001) | -0.331 (<.001) | 0.164 (<.001) | 0.160 (<.001) | -0.452 (<.001) | 0.137 (.001) | 0.179 (<.001) | 0.028 (.520) | 0.173 (<.001) |  |  |  |  |  |  |  |  |
| SDO (11) | 0.237 (<.001) | 0.322 (<.001) | -0.276 (<.001) | -0.043 (.314) | 0.250 (<.001) | 0.002 (.959) | -0.036 (.399) | -0.034 (.427) | -0.068 (.113) | -0.199 (<.001) |  |  |  |  |  |  |  |
| Gender (12) | 0.081 (.059) | -0.233 (<.001) | -0.138 (.001) | -0.134 (.002) | -0.005 (.913) | -0.039 (.366) | -0.101 (.018) | 0.188 (<.001) | -0.063 (.142) | 0.119 (.006) | -0.061 (.158) |  |  |  |  |  |  |
| Age (13) | -0.306 (<.001) | -0.151 (<.001) | 0.273 (<.001) | 0.124 (.004) | -0.187 (<.001) | 0.100 (.019) | 0.199 (<.001) | -0.029 (.499) | 0.143 (.001) | 0.343 (<.001) | 0.012 (.780) | -0.031 (.469) |  |  |  |  |  |
| Education (14) | -0.044 (.305) | -0.082 (.058) | 0.140 (.001) | 0.041 (.345) | -0.063 (.143) | -0.000 (.991) | -0.021 (.634) | 0.007 (.864) | 0.075 (.081) | -0.046 (.283) | -0.155 (<.001) | 0.008 (.854) | -0.123 (.004) |  |  |  |  |
|  | 1 | 2 | 3 | 4 | 5 | 6 | 7 | 8 | 9 | 10 | 11 | 12 | 13 | 14 | 15 | 16 | 17 |
| Employment (15) | -0.047 (.274) | -0.061 (.153) | -0.030 (.487) | -0.041 (.340) | -0.003 (.935) | -0.047 (.274) | -0.075 (.080) | -0.056 (.192) | -0.070 (.103) | 0.114 (.007) | -0.056 (.188) | 0.088 (.040) | 0.136 (.001) | -0.215 (<.001) |  |  |  |
| SES (16) | 0.054 (.205) | -0.024 (.582) | -0.161 (<.001) | -0.136 (.001) | 0.044 (.302) | -0.177 (<.001) | -0.034 (.428) | -0.002 (.964) | -0.170 (<.001) | 0.029 (.503) | -0.021 (.619) | 0.103 (.016) | -0.007 (.875) | -0.237 (<.001) | 0.267 (<.001) |  |  |
| Political Orient. (17) | -0.030 (.488) | 0.092 (.031) | 0.015 (.729) | 0.121 (.005) | -0.006 (.887) | 0.105 (.014) | 0.069 (.107) | 0.004 (.935) | 0.119 (.005) | 0.096 (.025) | 0.264 (<.001) | -0.052 (.228) | 0.126 (.003) | -0.057 (.190) | 0.010 (.819) | -0.012 (.775) |  |
| Income | 0.000 (.992) | -0.009 (.838) | 0.161 (<.001) | 0.017 (.709) | -0.034 (.445) | 0.054 (.222) | -0.024 (.593) | -0.041 (.357) | 0.076 (.087) | -0.045 (.311) | -0.084 (.057) | -0.106 (.018) | -0.151 (.001) | 0.269 (<.001) | -0.382 (<.001) | -0.479 (<.001) | 0.035 (.433) |

Note. L-PS: Legitimacy of Secret Power Scale. P-values appear in parentheses. Gender: 1 = male, 2 = female, Employment: 1 = Employed, 2 = Unemployed.

| Table Q. Correlations among all variables in Study 3 (Japan) | | | | | | | | | | | | | | | | | |
| --- | --- | --- | --- | --- | --- | --- | --- | --- | --- | --- | --- | --- | --- | --- | --- | --- | --- |
|  | 1 | 2 | 3 | 4 | 5 | 6 | 7 | 8 | 9 | 10 | 11 | 12 | 13 | 14 | 15 | 16 | 17 |
| L-SP (1) |  |  |  |  |  |  |  |  |  |  |  |  |  |  |  |  |  |
| Masculine Honor (2) | 0.062 (.137) |  |  |  |  |  |  |  |  |  |  |  |  |  |  |  |  |
| Democracy (3) | -0.295 (<.001) | 0.076 (.068) |  |  |  |  |  |  |  |  |  |  |  |  |  |  |  |
| Cooperation Legal Authorities (4) | -0.215 (<.001) | 0.152 (<.001) | 0.134 (.001) |  |  |  |  |  |  |  |  |  |  |  |  |  |  |
| Legal Cynicism (5) | 0.387 (<.001) | 0.133 (.001) | -0.226 (<.001) | -0.192 (<.001) |  |  |  |  |  |  |  |  |  |  |  |  |  |
| Legitimacy Police (6) | -0.207 (<.001) | 0.113 (.007) | 0.362 (<.001) | 0.210 (<.001) | -0.198 (<.001) |  |  |  |  |  |  |  |  |  |  |  |  |
| National Threat (7) | -0.383 (<.001) | 0.034 (.411) | 0.151 (<.001) | 0.232 (<.001) | -0.260 (<.001) | 0.147 (<.001) |  |  |  |  |  |  |  |  |  |  |  |
| Fear (8) | -0.358 (<.001) | 0.104 (.013) | 0.232 (<.001) | 0.211 (<.001) | -0.222 (<.001) | 0.214 (<.001) | 0.389 (<.001) |  |  |  |  |  |  |  |  |  |  |
| Anger (9) | -0.420 (<.001) | 0.146 (<.001) | 0.268 (<.001) | 0.365 (<.001) | -0.273 (<.001) | 0.197 (<.001) | 0.419 (<.001) | 0.667 (<.001) |  |  |  |  |  |  |  |  |  |
| Civic Honesty (10) | -0.425 (<.001) | -0.025 (.549) | 0.204 (<.001) | 0.174 (<.001) | -0.487 (<.001) | 0.180 (<.001) | 0.305 (<.001) | 0.225 (<.001) | 0.279 (<.001) |  |  |  |  |  |  |  |  |
| SDO (11) | 0.288 (<.001) | -0.015 (.717) | -0.157 (<.001) | -0.159 (<.001) | 0.252 (<.001) | -0.187 (<.001) | -0.199 (<.001) | -0.276 (<.001) | -0.289 (<.001) | -0.237 (<.001) |  |  |  |  |  |  |  |
| Gender (12) | -0.001 (.981) | -0.124 (.003) | -0.106 (.012) | -0.169 (<.001) | -0.102 (.015) | 0.052 (.215) | 0.042 (.323) | 0.152 (<.001) | 0.022 (.601) | 0.084 (.046) | -0.120 (.004) |  |  |  |  |  |  |
| Age (13) | -0.249 (<.001) | 0.027 (.520) | 0.260 (<.001) | 0.089 (.033) | -0.159 (<.001) | 0.095 (.024) | 0.248 (<.001) | 0.165 (<.001) | 0.290 (<.001) | 0.098 (.019) | -0.091 (.030) | -0.007 (.863) |  |  |  |  |  |
| Education (14) | -0.043 (.308) | -0.010 (.809) | 0.158 (<.001) | 0.036 (.395) | -0.111 (.008) | 0.087 (.039) | 0.030 (.467) | 0.031 (.463) | 0.024 (.568) | -0.012 (.770) | -0.028 (.507) | -0.090 (.032) | 0.008 (.840) |  |  |  |  |
|  | 1 | 2 | 3 | 4 | 5 | 6 | 7 | 8 | 9 | 10 | 11 | 12 | 13 | 14 | 15 | 16 | 17 |
| Employment (15) | -0.039 (.357) | -0.037 (.383) | 0.037 (.379) | -0.080 (.057) | -0.034 (.422) | 0.079 (.060) | 0.031 (.462) | 0.084 (.045) | -0.007 (.876) | 0.062 (.137) | -0.092 (.027) | 0.132 (.002) | 0.081 (.053) | -0.159 (<.001) |  |  |  |
| SES (16) | 0.007 (.865) | -0.119 (.004) | -0.125 (.003) | -0.104 (.013) | 0.057 (.170) | -0.228 (<.001) | 0.015 (.726) | -0.010 (.816) | -0.113 (.007) | -0.007 (.862) | 0.013 (.756) | 0.019 (.657) | -0.096 (.022) | -0.236 (<.001) | 0.149 (<.001) |  |  |
| Political Orient. (17) | 0.042 (.318) | 0.104 (.013) | 0.073 (.079) | 0.147 (<.001) | 0.013 (.749) | 0.033 (.433) | 0.001 (.984) | 0.045 (.286) | 0.075 (.072) | 0.000 (.993) | 0.087 (.038) | -0.166 (<.001) | -0.037 (.377) | -0.023 (.584) | -0.019 (.655) | -0.095 (.023) |  |
| Income | -0.066 (.144) | 0.085 (.061) | 0.050 (.267) | 0.177 (<.001) | -0.035 (.445) | 0.051 (.259) | -0.003 (.946) | 0.006 (.901) | 0.138 (.002) | 0.036 (.423) | 0.069 (.129) | -0.064 (.158) | 0.077 (.091) | 0.296 (<.001) | -0.299 (<.001) | -0.479 (<.001) | 0.094 (.038) |

Note. L-PS: Legitimacy of Secret Power Scale. P-values appear in parentheses. Gender: 1 = male, 2 = female, Employment: 1 = Employed, 2 = Unemployed.

**Measurement invariance of Legitimacy of the Police and Willingness to Cooperate with Legal Authorities (Study 3)**

We tested the measurement stability of the Legitimacy of the Police scale and participants’ Willingness to Cooperate with Legal Authorities. Our objective for this set of analyses was to achieve metric stability in order to compare relationships among the constructs across countries. The configural model of the Legitimacy of the Police scale achieved acceptable fit across the four countries, CFI = .94, RMSEA = .078, SRMR = .035, once Item 3 (“Police officers always treat people with respect”) was allowed to covary with Items 8 (“Police officers are excellent at communicating with people”) and 10 (“The explanations that police officers give for a stop are always fair”). The scale achieved full metric stability, ΔCFI = -.006.

A configural model had to be assumed for the measure of participants’ Willingness to Cooperate with Legal Authorities because the model was fully saturated (i.e., it only included three items). A model with all the factor loadings constrained to be the same across countries had an adequate fit, CFI = .99, RMSEA = .07, SRMR = .29, suggesting full metric invariance.

| Table R. Parameters for the covariates in the model predicting Willingness to Report Criminal Groups Activity across Countries (Study 3). | | | | | | |
| --- | --- | --- | --- | --- | --- | --- |
| **Predictors** | **β** | ***b*** | ***SE*** | ***z*** | ***p*** | **95% CI** |
| **United Kingdom** | | | | | | |
| Gender | -.138 | -.292 | .087 | -3.350 | = .001 | -.219 to -.057 |
| Age | -.007 | .001 | .003 | -.167 | = .867 | -.089 to .075 |
| SES | -.090 | -.054 | .028 | -1.972 | = .049 | -.180 to -.001 |
| Employment | -.019 | -.045 | .105 | -.428 | = .668 | -.104 to .067 |
| Income | -.089 | -.367 | .183 | -2.002 | = .045 | -.174 to -.003 |
| Education | .054 | .223 | .172 | 1.297 | = .195 | -.027 to .136 |
| Political Orientation | .130 | .068 | .023 | 2.970 | = .003 | .044 to .216 |
| **United States** | | | | | | |
| Gender | -.007 | -.014 | .076 | -.180 | = .857 | -.085 to .071 |
| Age | .048 | .003 | .003 | 1.098 | = .272 | -.038 to .134 |
| SES | -.107 | -.051 | .024 | -2.112 | = .035 | -.205 to -.009 |
| Employment | .007 | .013 | .102 | .130 | = .897 | -.095 to .108 |
| Income | .077 | .285 | .192 | 1.484 | = .138 | -.025 to .178 |
| Education | -.056 | -.187 | .165 | -1.132 | = .257 | -.153 to .041 |
| Political Orientation | .063 | .021 | .013 | 1.560 | = .119 | -.017 to .142 |
| **Italy** | | | | | | |
| Gender | -.064 | -.120 | .086 | -1.385 | = .166 | -.154 to .027 |
| Age | .023 | .002 | .003 | .460 | = .646 | -.075 to .121 |
| **Predictors** | **β** | ***B*** | ***SE*** | ***Z*** | ***P*** | **95% CI** |
| SES | -.030 | -.018 | .030 | -.599 | = .549 | -.128 to .068 |
| Employment | .020 | .042 | .101 | .413 | = .680 | -.076 to .116 |
| Income | .084 | .321 | .190 | 1.693 | = .090 | -.013 to.182 |
| Education | .095 | .342 | .153 | 2.232 | = .026 | .013 to.178 |
| Political Orientation | .016 | .006 | .017 | .330 | = .741 | -.080 to.112 |
| **Japan** | | | | | | |
| Gender | -.161 | -.319 | .092 | -3.469 | = .001 | -.250 to -.071 |
| Age | .052 | .004 | .003 | 1.167 | = .243 | -.035 to .138 |
| SES | .057 | .031 | .032 | .987 | = .324 | -.056 to .170 |
| Employment | -.086 | -.197 | .114 | -1.724 | = .085 | -.183 to .011 |
| Income | .153 | .565 | .210 | 2.691 | = .007 | .044 to .262 |
| Education | -.049 | -.148 | .143 | -1.041 | = .298 | -.141 to .043 |
| Political Orientation | .135 | .091 | .032 | 2.825 | = .005 | .042 to .228 |

Note. Gender: 1 = male, 2 = female; Employment: 1 = Unemployed, 2 = Employed; SES = higher values indicate higher position on the socio-economic ladder; Income and education Income and Education were percentile ranks (higher values indicate more education and higher income).

# Section D: Testing a Shorter Version of the L-SP Scale (L-SPs)

In addition to the 20-item L-SP scale described in the main manuscript, we explored the feasibility of a shorter version comprising ten items for use in contexts where brevity is essential (L-SPs). This abbreviated version was derived based on the highest factor loadings obtained in Study 2, which collected data for 20 items (See Table E above). We retained the first eight and two reversed items to safeguard against response biases. The short scale demonstrates a good overlap with other constructs and shows excellent psychometric indices. Below we employ tables to summarise the analyses employing L-SPs.

Table S. L-SPs

| L-SP1 | Criminal groups provide justice effectively in the community |
| --- | --- |
| L-SP2 | Criminal groups provide protection in an efficient way in the community |
| L-SP3 | Criminal groups are sometimes necessary to maintain order in the community |
| L-SP5 | Criminal groups protect individuals' rights |
| L-SP10_R | Criminal groups’ activities can only have negative consequences for people |
| L-SP19 | Criminal groups play a positive role in promoting economic development in disadvantaged communities |
| L-SP22_R | Criminal groups harm the economy of the community |
| L-SP27 | Criminal groups are necessary to get things done in the community |
| L-SP28 | Criminal groups can sometimes be trusted to act in the community's best interests |
| L-SP32 | Criminal groups are effective at maintaining social stability |

Employing data from Study 2, we tested the measurement model using the ten items summarized in Table S. The fit indices were excellent for a model employing covariates among reversed items and a model without covariates (Table T). Standardized factor loadings are summarized in Table U. L-SPs’ mean was *M* = 2.342 (*SD* = 1.06).

Table T. Measurement models for L-SPs.

|  | without covariances between reversed items | with covariances between reversed items |
| --- | --- | --- |
| CFI | 0.982 | 0.990 |
| RMSEA | 0.055 | 0.041 |
| SRMR | 0.030 | 0.023 |

Table U. Standardised factor loadings for L-SPs’ items.

| Item | without covariances between reversed items | with covariances between reversed items |
| --- | --- | --- |
| LSP1 | 0.770 | 0.771 |
| LSP2 | 0.783 | 0.784 |
| LSP3 | 0.822 | 0.822 |
| LSP5 | 0.757 | 0.758 |
| LSP10_R | 0.623 | 0.614 |
| LSP19 | 0.738 | 0.737 |
| LSP22_R | 0.545 | 0.533 |
| LSP27 | 0.796 | 0.797 |
| LSP28 | 0.780 | 0.781 |
| LSP32 | 0.796 | 0.798 |

As shown in Tables V and W, the L-SPs scale’s correlations with other constructs and demographic variables were similar to those of the longer scale version.

Table V. Correlations of L-SPs with other constructs (refer to the main article for details)

| Variable | r | 95% CI | df | p-value |
| --- | --- | --- | --- | --- |
| Extrajudicial violence | 0.314 | 0.231 to 0.392 | 485 | < .001 |
| Masculine Honor | 0.330 | 0.249 to 0.407 | 485 | < .001 |
| Attitudes towards Democracy | -0.258 | -0.339 to -0.173 | 485 | < .001 |
| Willingness to cooperate with legal author. | -0.337 | -0.413 to -0.255 | 485 | < .001 |
| General Aggressive Tendencies | 0.165 | 0.077 to 0.25 | 485 | < .001 |
| Legal Cynicism | 0.398 | 0.321 to 0.471 | 485 | < .001 |
| Police Legitimacy | -0.200 | -0.284 to -0.113 | 485 | < .001 |
| National Threat from Criminal Groups | -0.385 | -0.459 to -0.307 | 484 | < .001 |
| Fear | -0.170 | -0.255 to -0.082 | 485 | < .001 |

Table W. Correlations of L-SPs with demographic variables (refer to the main article for details)

| Variable | r | 95% CI | df | p-value |
| --- | --- | --- | --- | --- |
| Social Desirability | -0.138 | -0.225 to -0.05 | 485 | 0.002 |
| Gender | -0.031 | -0.12 to 0.059 | 479 | 0.500 |
| Age | -0.245 | -0.327 to -0.16 | 485 | 0.000 |
| Education | 0.015 | -0.075 to 0.104 | 481 | 0.746 |
| Employment | -0.053 | -0.142 to 0.037 | 476 | 0.246 |
| Subjective Socio Economic Status | 0.085 | -0.003 to 0.173 | 485 | 0.059 |
| Political Orientation | -0.018 | -0.107 to 0.071 | 485 | 0.689 |

We tested a latent model in which L-SPs predicted participants’ willingness to cooperate with legal authorities, controlling for fear and perceived police legitimacy. The model is summarized in Table Y.

Table Y. Structural equation model employing L-SPs to predict participants’ willingness to cooperate with legal authorities (refer to the main article for details)

| Predictors | B | SE | Standardized Path | z | p | 95% CI |
| --- | --- | --- | --- | --- | --- | --- |
| L-SPs | -0.339 | 0.057 | -0.297 | -5.912 | 0.000 | -0.451 to -0.226 |
| Police Leg. | 0.343 | 0.073 | 0.256 | 4.685 | 0.000 | 0.2 to 0.487 |
| Fear | 0.048 | 0.032 | 0.042 | 1.487 | 0.137 | -0.015 to 0.11 |
| Age | 0.005 | 0.004 | 0.004 | 1.327 | 0.185 | -0.002 to 0.013 |
| Gender | -0.036 | 0.101 | -0.032 | -0.357 | 0.721 | -0.233 to 0.161 |
| Employment | 0.067 | 0.124 | 0.059 | 0.539 | 0.590 | -0.176 to 0.309 |
| Education | 0.084 | 0.037 | 0.074 | 2.253 | 0.024 | 0.011 to 0.156 |
| Subj. Socio Econ. | -0.098 | 0.038 | -0.086 | -2.572 | 0.010 | -0.172 to -0.023 |
| Political Orient. | 0.005 | 0.023 | 0.004 | 0.206 | 0.837 | -0.041 to 0.05 |

The measurement model of L-SPs was further tested and confirmed across the four countries of Italy, the UK, Japan, and the US employing the data from Study 3 (see Tables Z1-Z2).

Table Z1. Measurement models for L-SPs across countries without covariances among reversed items

|  | CFI | RMSEA | SRMR |
| --- | --- | --- | --- |
| United States | 0.986 | 0.050 | 0.035 |
| United Kingdom | 0.987 | 0.045 | 0.030 |
| Italy | 0.989 | 0.041 | 0.028 |
| Japan | 0.971 | 0.066 | 0.037 |

Table Z2. Measurement models for L-SPs across countries with covariances among reversed items

|  | CFI | RMSEA | SRMS |
| --- | --- | --- | --- |
| United States | 1.000 | 0.000 | 0.017 |
| United Kingdom | 0.998 | 0.019 | 0.020 |
| Italy | 0.995 | 0.029 | 0.020 |
| Japan | 0.983 | 0.051 | 0.028 |

Standardized factor loadings for L-SPs’ items of the models with and without covariances are summarized in Tables A1-A2 below, for each of the four countries.

Table A1. Standardized factor loading L-SPs model without covariances among reversed items

|  | US | UK | IT | JA |
| --- | --- | --- | --- | --- |
| L-SP1 | 0.810 | 0.804 | 0.684 | 0.708 |
| L-SP2 | 0.849 | 0.785 | 0.708 | 0.747 |
| L-SP3 | 0.786 | 0.793 | 0.778 | 0.789 |
| L-SP5 | 0.799 | 0.759 | 0.839 | 0.727 |
| L-SP10_R | 0.516 | 0.575 | 0.403 | 0.510 |
| L-SP19 | 0.785 | 0.782 | 0.756 | 0.772 |
| L-SP22_R | 0.468 | 0.548 | 0.416 | 0.577 |
| L-SP27 | 0.768 | 0.804 | 0.862 | 0.733 |
| L-SP28 | 0.807 | 0.774 | 0.790 | 0.797 |
| L-SP32 | 0.780 | 0.757 | 0.783 | 0.800 |

Table A2. Standardized factor loading L-SPs model with covariances among reversed items

|  | US | UK | IT | JA |
| --- | --- | --- | --- | --- |
| L-SP1 | 0.811 | 0.804 | 0.684 | 0.709 |
| L-SP2 | 0.849 | 0.786 | 0.707 | 0.748 |
| L-SP3 | 0.787 | 0.794 | 0.778 | 0.789 |
| L-SP5 | 0.799 | 0.759 | 0.839 | 0.727 |
| L-SP10_R | 0.507 | 0.564 | 0.397 | 0.496 |
| L-SP19 | 0.785 | 0.782 | 0.756 | 0.773 |
| L-SP22_R | 0.457 | 0.536 | 0.410 | 0.566 |
| L-SP27 | 0.769 | 0.805 | 0.863 | 0.733 |
| L-SP28 | 0.808 | 0.775 | 0.791 | 0.799 |
| L-SP32 | 0.780 | 0.758 | 0.783 | 0.801 |

L-SPs’ achieved full scalar invariance regardless of whether covariances among reversed items were included in the model or not. The models are summarised in Tables B1-B2 below.

Table B1. Measurement models for L-SPs model with and without covariances among reversed items

|  | with covariances among reversed items | | |
| --- | --- | --- | --- |
|  | CFI | RMSEA | SRMS |
| Configural | 0.994 | 0.030 | 0.019 |
| Metric | 0.992 | 0.033 | 0.040 |
| Scalar | 0.982 | 0.046 | 0.048 |
|  | without covariances among reversed items | | |
| Configural | 0.983 | 0.051 | 0.030 |
| Metric | 0.981 | 0.051 | 0.046 |
| Scalar | 0.971 | 0.058 | 0.052 |

Table B2. Measurement invariance tests for L-SPs models with and without covariances among reversed items

|  | with covariances among reversed items | | |
| --- | --- | --- | --- |
|  | CFI | ΔGamma hat | ΔNCI |
| Configural - Metric | 0.002 | 0.002 | 0.008 |
| Metric - Scalar | 0.010 | 0.007 | 0.027 |
|  | without covariances among reversed items | | |
| Configural - Metric | 0.003 | 0.002 | 0.008 |
| Metric - Scalar | 0.010 | 0.007 | 0.025 |

Correlations between L-SPs and other variables are summarized in tables C1-C4 below.

###

Table C1. Japan: Correlations between the L-SP scale and the other variables in Study 3(refer to the main article for details)

|  | r | 95% CI | df | p-value |
| --- | --- | --- | --- | --- |
| Masculine Honor Ideology | 0.049 | -0.033 to 0.131 | 570 | 0.239 |
| Democracy | -0.292 | -0.366 to -0.216 | 570 | 0.000 |
| Willingness to cooperate with legal auth. | -0.209 | -0.286 to -0.129 | 570 | 0.000 |
| Legal Cynicism | 0.367 | 0.294 to 0.436 | 570 | 0.000 |
| Police Legitimacy | -0.190 | -0.267 to -0.109 | 570 | 0.000 |
| National Threat | -0.375 | -0.443 to -0.302 | 570 | 0.000 |
| Fear | -0.353 | -0.423 to -0.279 | 570 | 0.000 |
| Anger | -0.413 | -0.479 to -0.343 | 570 | 0.000 |
| Civic Honesty | -0.405 | -0.472 to -0.335 | 570 | 0.000 |
| Social Dominance Orientation | 0.272 | 0.194 to 0.346 | 570 | 0.000 |

Table C2. Italy: Correlations between the L-SP scale and the other variables in Study 3 (refer to the main article for details)

|  | r | 95% CI | df | p-value |
| --- | --- | --- | --- | --- |
| Masculine Honor Ideology | 0.321 | 0.246 to 0.392 | 578 | 0.000 |
| Democracy | -0.302 | -0.374 to -0.226 | 578 | 0.000 |
| Willingness to cooperate with legal auth. | -0.293 | -0.366 to -0.217 | 578 | 0.000 |
| Legal Cynicism | 0.361 | 0.288 to 0.43 | 578 | 0.000 |
| Police Legitimacy | -0.196 | -0.273 to -0.116 | 578 | 0.000 |
| National Threat | -0.482 | -0.542 to -0.417 | 578 | 0.000 |
| Fear | -0.074 | -0.155 to 0.007 | 578 | 0.075 |
| Anger | -0.343 | -0.413 to -0.269 | 578 | 0.000 |
| Civic Honesty | -0.490 | -0.549 to -0.425 | 578 | 0.000 |
| Social Dominance Orientation | 0.373 | 0.301 to 0.441 | 578 | 0.000 |

###

Table C3. USA: Correlations between the L-SP scale and the other variables in Study 3 (refer to the main article for details)

|  | r | 95% CI | df | p-value |
| --- | --- | --- | --- | --- |
| Masculine Honor Ideology | 0.225 | 0.147 to 0.3 | 588 | 0 |
| Democracy | -0.260 | -0.334 to -0.183 | 588 | 0 |
| Willingness to cooperate with legal auth. | -0.247 | -0.322 to -0.17 | 588 | 0 |
| Legal Cynicism | 0.398 | 0.327 to 0.463 | 588 | 0 |
| Police Legitimacy | -0.185 | -0.262 to -0.106 | 588 | 0 |
| National Threat | -0.410 | -0.475 to -0.341 | 588 | 0 |
| Fear | -0.160 | -0.238 to -0.081 | 588 | 0 |
| Anger | -0.335 | -0.405 to -0.262 | 588 | 0 |
| Civic Honesty | -0.390 | -0.456 to -0.319 | 588 | 0 |
| Social Dominance Orientation | 0.211 | 0.133 to 0.287 | 588 | 0 |

###

Table C4. UK: Correlations between the L-SP scale and the other variables in Study 3 (refer to the main article for details)

|  | r | 95% CI | df | p-value |
| --- | --- | --- | --- | --- |
| Masculine Honor Ideology | 0.275 | 0.196 to 0.351 | 545 | 0.000 |
| Democracy | -0.321 | -0.394 to -0.244 | 545 | 0.000 |
| Willingness to cooperate with legal auth. | -0.347 | -0.419 to -0.271 | 545 | 0.000 |
| Legal Cynicism | 0.409 | 0.336 to 0.476 | 545 | 0.000 |
| Police Legitimacy | -0.150 | -0.231 to -0.067 | 545 | 0.000 |
| National Threat | -0.402 | -0.47 to -0.33 | 545 | 0.000 |
| Fear | -0.092 | -0.175 to -0.009 | 545 | 0.031 |
| Anger | -0.353 | -0.424 to -0.277 | 545 | 0.000 |
| Civic Honesty | -0.375 | -0.445 to -0.301 | 545 | 0.000 |
| Social Dominance Orientation | 0.214 | 0.132 to 0.292 | 545 | 0.000 |

Tables D1-D4 summarize the correlations between L-SPs and the demograpgic variables across countries.

Table D1. Japan: Correlations between L-SP and demographic variables in Study 3 (refer to the main article for details)

|  | r | 95% CI | df | p-value |
| --- | --- | --- | --- | --- |
| Gender | 0.006 | -0.076 to 0.088 | 567 | 0.884 |
| Age | -0.249 | -0.325 to -0.171 | 570 | 0.000 |
| Education | -0.061 | -0.142 to 0.022 | 568 | 0.149 |
| Employment | -0.021 | -0.102 to 0.062 | 570 | 0.624 |
| Subjective Socio Econ. Status | 0.014 | -0.068 to 0.095 | 570 | 0.746 |
| Political Orientation | 0.051 | -0.031 to 0.133 | 570 | 0.219 |
| Income | -0.086 | -0.173 to 0.003 | 486 | 0.058 |

Table D2. Italy: Correlations between L-SP and demographic variables in Study 3 (refer to the main article for details)

| Variable | r | 95% CI | df | p-value |
| --- | --- | --- | --- | --- |
| Gender | -0.076 | -0.157 to 0.006 | 575 | 0.068 |
| Age | -0.198 | -0.275 to -0.118 | 578 | 0.000 |
| Education | -0.046 | -0.127 to 0.036 | 574 | 0.273 |
| Employment | -0.020 | -0.101 to 0.062 | 578 | 0.635 |
| Subjective Socio Econ. Status | -0.078 | -0.159 to 0.003 | 578 | 0.060 |
| Political Orientation | 0.132 | 0.051 to 0.211 | 578 | 0.001 |
| Income | -0.045 | -0.131 to 0.041 | 519 | 0.304 |

Table D3. USA: Correlations between L-SP and demographic variables in Study 3 (refer to the main article for details)

| Variable | r | 95% CI | df | p-value |
| --- | --- | --- | --- | --- |
| Gender | 0.025 | -0.056 to 0.106 | 587 | 0.546 |
| Age | -0.336 | -0.406 to -0.262 | 588 | 0.000 |
| Education | -0.054 | -0.134 to 0.027 | 588 | 0.193 |
| Employment | -0.067 | -0.147 to 0.014 | 588 | 0.106 |
| Subjective Socio Econ. Status | -0.062 | -0.142 to 0.019 | 588 | 0.132 |
| Political Orientation | 0.083 | 0.003 to 0.163 | 588 | 0.043 |
| Income | -0.052 | -0.133 to 0.03 | 576 | 0.214 |

Table D4. UK: Correlations between L-SP and demographic variables in Study 3 (refer to the main article for details)

| Variable | r | 95% CI | df | p-value |
| --- | --- | --- | --- | --- |
| Gender | 0.076 | -0.009 to 0.159 | 538 | 0.079 |
| Age | -0.285 | -0.36 to -0.206 | 545 | 0.000 |
| Education | -0.046 | -0.13 to 0.039 | 538 | 0.287 |
| Employment | -0.041 | -0.124 to 0.043 | 545 | 0.337 |
| Subjective Socio Econ. Status | 0.063 | -0.021 to 0.146 | 545 | 0.143 |
| Political Orientation | -0.048 | -0.131 to 0.036 | 545 | 0.264 |
| Income | -0.013 | -0.1 to 0.074 | 508 | 0.772 |

Finally, we employed L-SPs in a latent model to predict participants’ willingness to cooperate with legal authorities across countries over and beyond fear and perceived police legitimacy. The analyses replicated the tests conducted in the main article. Below, in Table E1, we summarize the parameters for a model in which only the path between fear and participants’ willingness to cooperate was constrained to be equal across countries.

Table E1. Parameters for the model predicting Willingness to Report Criminal Groups Activity across Countries (Study 3)

| Predictors | B | SE | Standardized Path | z | p | 95% CI |
| --- | --- | --- | --- | --- | --- | --- |
|  |  |  | **Italy** |  |  |  |
| L-SPs | -0.214 | 0.042 | -0.233 | -5.148 | 0.000 | -0.296 to -0.133 |
| Police Leg. | 0.319 | 0.070 | 0.228 | 4.543 | 0.000 | 0.182 to 0.457 |
| Fear | 0.073 | 0.015 | 0.078 | 4.812 | 0.000 | 0.043 to 0.103 |
| Gender | -0.118 | 0.087 | -0.126 | -1.367 | 0.172 | -0.288 to 0.051 |
| Age | 0.002 | 0.003 | 0.002 | 0.541 | 0.588 | -0.005 to 0.009 |
| Subj. Socio Eco | -0.019 | 0.030 | -0.020 | -0.633 | 0.527 | -0.078 to 0.04 |
| Employment | 0.048 | 0.101 | 0.051 | 0.476 | 0.634 | -0.15 to 0.247 |
| Income | 0.336 | 0.190 | 0.359 | 1.767 | 0.077 | -0.037 to 0.71 |
| Education | 0.353 | 0.154 | 0.377 | 2.299 | 0.022 | 0.052 to 0.655 |
| Pol. Or. | 0.005 | 0.017 | 0.005 | 0.287 | 0.774 | -0.029 to 0.039 |
|  |  |  | **Japan** |  |  |  |
| L-SPs | -0.121 | 0.046 | -0.121 | -2.643 | 0.008 | -0.211 to -0.031 |
| Police Leg. | 0.245 | 0.069 | 0.178 | 3.576 | 0.000 | 0.111 to 0.38 |
| Fear | 0.073 | 0.015 | 0.074 | 4.812 | 0.000 | 0.043 to 0.103 |
| Gender | -0.320 | 0.092 | -0.322 | -3.476 | 0.001 | -0.5 to -0.14 |
| Age | 0.004 | 0.003 | 0.004 | 1.164 | 0.244 | -0.003 to 0.01 |
| Subj. Socio Eco | 0.031 | 0.032 | 0.032 | 0.993 | 0.321 | -0.031 to 0.093 |
| Employment | -0.196 | 0.114 | -0.197 | -1.717 | 0.086 | -0.419 to 0.028 |
| Income | 0.562 | 0.210 | 0.567 | 2.676 | 0.007 | 0.15 to 0.974 |
| Education | -0.153 | 0.142 | -0.154 | -1.072 | 0.284 | -0.432 to 0.126 |
| Pol. Or. | 0.092 | 0.032 | 0.093 | 2.852 | 0.004 | 0.029 to 0.155 |
|  |  |  | **United States** |  |  |  |
| L-SPs | -0.106 | 0.028 | -0.153 | -3.733 | 0.000 | -0.161 to -0.05 |
| Police Leg. | 0.448 | 0.055 | 0.430 | 8.158 | 0.000 | 0.34 to 0.556 |
| Fear | 0.073 | 0.015 | 0.076 | 4.812 | 0.000 | 0.043 to 0.103 |
| Gender | -0.017 | 0.076 | -0.018 | -0.222 | 0.825 | -0.167 to 0.133 |
| Age | 0.003 | 0.003 | 0.004 | 1.175 | 0.240 | -0.002 to 0.009 |
| Subj. Socio Eco | -0.049 | 0.024 | -0.052 | -2.064 | 0.039 | -0.096 to -0.002 |
| Employment | 0.012 | 0.102 | 0.012 | 0.116 | 0.907 | -0.188 to 0.212 |
| Income | 0.285 | 0.192 | 0.297 | 1.481 | 0.139 | -0.092 to 0.661 |
| Education | -0.188 | 0.166 | -0.196 | -1.133 | 0.257 | -0.512 to 0.137 |
| Pol. Or. | 0.021 | 0.013 | 0.022 | 1.590 | 0.112 | -0.005 to 0.048 |
|  |  |  | **United Kingdom** |  |  |  |
| L-SPs | -0.243 | 0.042 | -0.273 | -5.789 | 0.000 | -0.325 to -0.161 |
| Police Leg. | 0.477 | 0.069 | 0.365 | 6.897 | 0.000 | 0.342 to 0.613 |
| Fear | 0.073 | 0.015 | 0.069 | 4.812 | 0.000 | 0.043 to 0.103 |
| Gender | -0.296 | 0.087 | -0.280 | -3.386 | 0.001 | -0.467 to -0.125 |
| Age | 0.000 | 0.003 | 0.000 | -0.004 | 0.997 | -0.006 to 0.006 |
| Subj. Socio Eco | -0.054 | 0.028 | -0.051 | -1.947 | 0.052 | -0.108 to 0 |
| Employment | -0.036 | 0.104 | -0.035 | -0.350 | 0.726 | -0.241 to 0.168 |
| Income | -0.368 | 0.183 | -0.349 | -2.010 | 0.044 | -0.727 to -0.009 |
| Education | 0.231 | 0.173 | 0.219 | 1.339 | 0.181 | -0.107 to 0.57 |
| Pol. Or. | 0.066 | 0.023 | 0.063 | 2.870 | 0.004 | 0.021 to 0.111 |
